# *Clostridioides difficile* infection, recurrence and the associated healthcare consumption in Sweden between 2006 and 2019: a population-based cohort study

**DOI:** 10.1186/s12879-024-09364-3

**Published:** 2024-05-03

**Authors:** Annelies Boven, Johanna Simin, Fredrik L. Andersson, Erika Vlieghe, Steven Callens, Zangin Zeebari, Lars Engstrand, Nele Brusselaers

**Affiliations:** 1 Centre for Translational Microbiome Research (CTMR), Department of Microbiology, Tumour and Cell Biology, Karolinska Institutet, Solnavägen 9, 171 65 Stockholm, Sweden; 2 Department of Family Medicine and Population Health, Antwerp University, Antwerp, Belgium; 3 Global Health Economics & Outcomes Research at Ferring Pharmaceuticals, Copenhagen, Denmark; 4 General Internal Medicine, Antwerp University Hospital, Antwerp, Belgium; 5 General Internal Medicine, Department of Internal Medicine and Paediatrics, Ghent University, Ghent, Belgium; 6 Department of Economics, Finance, Statistics and Informatics, Jönköping University, Jönköping, Sweden; 7 Department of Global Public Health, Karolinska Institute, Stockholm, Sweden; 8 Department of Public Health and Primary Care, Ghent University, Ghent, Belgium

**Keywords:** *Clostridioides difficile*, Healthcare consumption, Health-economic, Burden, Real world evidence

## Abstract

**Background:**

*Clostridioides difficile* infection (CDI) causes a major burden to individuals and society, yet the impact may vary depending on age, sex, underlying comorbidities and where CDI was acquired (hospital or community).

**Methods:**

This Swedish nationwide population-based cohort study (2006–2019) compared all 43,150 individuals with CDI to their 355,172 matched controls (first year and entire follow-up). Negative binomial regression models compared the cumulated length of stay, number of in-hospital admissions, outpatient visits and prescriptions after the first CDI episode expressed as incidence rate ratios (IRR) and 95% confidence intervals for the entire follow-up.

**Results:**

Overall, 91.6% of CDI cases were hospital acquired, and 16.8% presented with recurrence(s); 74.8%of cases were ≥ 65 years and 54.2% were women. Compared to individuals without CDI, in-hospital stay rates were 18.01 times higher after CDI (95% CI 17.40–18.63, first-year: 27.4 versus 1.6 days), 9.45 times higher in-hospital admission (95% CI 9.16–9.76, first-year: 2.6 versus 1.3 hospitalisations), 3.94 times higher outpatient visit (95% CI 3.84–4.05, first-year: 4.0 versus 1.9 visits) and 3.39 times higher dispensed prescriptions rates (95% CI 3.31–3.48, first-year: 25.5 versus 13.7 prescriptions). For all outcomes, relative risks were higher among the younger (< 65 years) than the older (≥ 65 years), and in those with fewer comorbidities, but similar between sexes. Compared to those without recurrence, individuals with recurrence particularly showed a higher rate of hospital admissions (IRR = 1.18, 95% 1.12–1.24). Compared to community-acquired CDI, those with hospital-acquired CDI presented with a higher rate of hospital admissions (IRR = 7.29, 95% CI 6.68–7.96) and a longer length of stay (IRR = 7.64, 95% CI 7.07–8.26).

**Conclusion:**

CDI was associated with increased health consumption in all affected patient groups. The majority of the CDI burden could be contributed to hospital-acquired CDI (~ 9/10), older patients (~ 3/4) and those with multiple comorbidities (~ 6/10 Charlson score ≥ 3), with 1/5 of the total CDI burden contributed to individuals with recurrence. Yet, relatively speaking the burden was higher among the younger and those with fewer comorbidities, compared to their peers without CDI.

**Supplementary Information:**

The online version contains supplementary material available at 10.1186/s12879-024-09364-3.

## Background

*Clostridioides difficile* infection (CDI) is a global problem, as one of the most prevalent healthcare-associated infections [1–5]. CDI morbidity and mortality are high, with an estimated 3–sevenfold times higher mortality than matched controls without CDI in Sweden, and 2.7 times higher in a Latin American study [6, 7]. A global meta-analyses estimated a CDI rated of 2.4 per 1000 admissions, and around 11.1/1000 for admissions at intensive care units [5]. Approximately 10–20% report at least one recurrence, or even up to 64% of hospital-acquired CDI, according to a large meta-analysis [2].

Based on two systematic reviews on the health-economic aspects of CDI, the additional cost of a CDI episode is approximately 24,000 USD, [8] while a recurrence could cost up to 82,000 USD extra, [9] with hospitalisation and duration of stay being the main cost drivers. A recent study estimated that one hospitalisation day at general wards costs 412€ in Northern Europe, and almost 2000€ in an intensive care unit [10]. Several systematic reviews discovered that important risk factors for increased length of stay and number of admissions, i.e. essential contributors to the healthcare consumption, are hospital-onset CDI, recurrent CDI and underlying comorbidities [8–11]. It is complicated to disentangle the effect of comorbidities and CDI on healthcare consumption, as the presence of comorbidities is linked to increased healthcare needs including outpatient visits and prescriptions for (maintenance) drug use. Chronic comorbidities are also associated with a higher likelihood of CDI, while CDI itself may result in a health decline and therefore higher healthcare needs related to these comorbidities [8]. Nevertheless, many previous studies did not adjust their healthcare burden results for comorbidities [8, 9].

Even though the economic burden of CDI is estimated to be high, large nationwide and population-based studies investigating the cost burden are sparse and variation is large [11–16]. Previous studies have mainly been conducted in hospital settings and on older populations, with relatively highly heterogeneous costing methods, hampering the generalizability of the results [8–11, 17]. Only few studies also investigated community-onset CDI which appears to contribute to a lower burden than hospital-acquired CDI; with a US study estimating the respective CDI attributable costs as 8,222 USD and 14,257 USD per patient [18, 19].

The aim of our current study is to achieve an informed estimate of the overall health care consumption directly or indirectly associated with hospital- and community-acquired CDI and recurrence in a Swedish nationwide and population-based matched cohort study. We compared all individuals with CDI to matched individuals without CDI, and compared the burden in individuals with and without CDI recurrence; while assessing the impact of age, origin of CDI (community or healthcare onset) and chronic comorbidities.

## Methods

### Definition of CDI

CDI was defined by the ICD-10-SE code A04.7 (“Enterocolitis caused by *C. difficile*”, the only CDI-specific code), ascertained from the Swedish in- and outpatient care registry. For individuals with several CDI episodes the date of the first episode was applied to define cohort entry. Recurrent CDI was defined as a CDI episode occurring within eight weeks from the start of the previous CDI episode [20]. CDI episodes were further classified into likely hospital-/community-acquired and of unknown origin, defined as a CDI diagnosis during or within 4 weeks after latest in-hospital admission (hospital-acquired), more than 12 weeks after latest hospital admission (community-acquired) and between 4–12 after latest hospital admission (unknown origin). History of CDI was classified by the presence of a CDI episode before the start of the study (from 1997 when the ICD-10 was introduced, up until 2005).

### Study base

This study was part of a larger, nationwide population-based study that aimed to assess multiple outcomes associated with CDI and recurrence (rCDI) among a Swedish cohort including virtually all residents with a recorded CDI diagnosis (*N* = 43,150) between January 2006 and December 2019 on the National Patient Registry [6, 21, 22]. The source cohort included all Swedish individuals diagnosed with the broader ICD-10 code A04, randomly matched to controls on year of birth and sex [6]. Population-controls were randomly selected from the Prescribed Drug Registry (full population coverage from July 2005 and onwards [23]). All received at least one dispensed prescription (of any drug) since the start of this registry (i.e., during a 15-year period which approximates the entire Swedish population). For this larger project, the controls were matched to the cases based on sex and year of birth (on 10:1 ratio) and ascertained not to have a CDI diagnosis during or before the study period based on the National Patient Registry (data from 1997 and onwards). For this project, we only included those with the specific CDI code (A04.7), and excluded all individuals with other A04 codes (*n* = 26,850) and their corresponding controls (*n* = 268,500). We also excluded all controls who died before their corresponding case had the first CDI episode (*n* = 76,348), yet this should not have affected the results drastically as all cases initially had 10 controls [6]. All data were linked by the National Board of Health and Welfare using the unique personal identifier of each Swedish resident [24]. The Swedish Ethical Review Authority approved the study (2020–02454) without the need for an informed consent due to the registry-based nature of the study.

### Outcome ascertainment

Primary outcomes were the cumulated length of stay (LOS), number of in-hospital admissions, number of outpatient care visits and cumulative use of prescription drugs within one year after the first CDI episode; and furthermore, during the whole study period. The day of in-hospital admission was defined as hospital day zero. The healthcare consumption related outcomes were ascertained from the National Patient Registry. Cumulative drug exposure was identified from the outpatient care Drug Registry. The date of the first CDI episode of the matched case was used as a proxy date for age at first CDI episode of the controls.

### Covariates

Considered covariates were age, sex, place of birth (Nordic or non-Nordic), Charlson Comorbidity Index (CCI) scores, underlying inflammatory bowel disease (IBD), underlying haematological diseases, CDI recurrence (within 8 weeks of a first CDI diagnosis), and were all obtained from the nationwide Patient Registries (for specific ICD-coding see Additional Table 1) apart from any malignancy (applied for the CCI score), which was obtained from the Swedish Cancer Registry [25]. Additional confounders were ever-use of antibiotics, aspirin, H_2_-receptor antagonists (H2RAs), non-steroidal anti-inflammatory drugs (NSAIDs) and proton pump inhibitors (PPIs) as they may affect the risk of CDI and CDI recurrence; [21] based on the Prescribed Drug Registry, before onset of the first CDI (Additional Table 2). Data on the region of birth (Nordic/non-Nordic) was combined from Patient Registry, Cancer Registry and Causes of Death Registry.

### Statistical analyses

Negative binominal regression models (used for count data) were utilized to assess the association of CDI with length of stay (LOS), number of admissions, number of outpatient care visits and cumulative number of dispensed prescriptions. Poisson modelling was not possible because the mean–variance equality assumption was not met due to overdispersion.

In these analyses individuals with CDI were compared to those without CDI, and rCDI to non-rCDI, providing incidence rate ratios (IRRs) with 95% confidence intervals (CIs) for the entire study period. Person-years were applied as offset in the models, thus considering time at risk (including censoring for death and end of study period). All individuals were followed-up from the initial CDI episode (or the proxy date for the matched controls), to death or end of study (December 2019), whichever occurred first. In the recurrence models’ individuals with CDI were followed-up from the first date of the second CDI episode, i.e., the recurrence. Besides the matching variables, models were adjusted for CCI score (continuous scale, avoiding information loss), IBD, haematological disease, region of birth (Nordic/ non-Nordic), and use of prescription drugs. Stratified analyses were conducted for recurrence, age-groups (categorized as < 65 and ≥ 65 years) and CCI score (categorized as 0, 1, 2, 3, 4, and ≥ 5). Analyses comparing rCDI to non-rCDI were further adjusted for CDI-history.

To assess a potential dynamic effect and the robustness of our results, descriptive analyses were conducted cross-sectionally (one year after CDI) and longitudinally (for the entire study period).

All analyses were conducted on STATA MP 14.2 and the significance level was fixed at 5%.

## Results

The final cohort included 43,150 individuals with and 355,172 individuals without at least one CDI episode (Table 1). Of the 43,150 cases, 16.8% (*N* = 7,251) had a recurrent CDI episode; and 91.6% (*N* = 39,536) was hospital-acquired (Table 1).

**Table 1:** Balance Table presenting demographical and clinical characteristics of all individuals with *Clostridioides difficile* infection (CDI), by recurrence status; and their matched controls (2006–2019)

	**All CDI *N* = 43,150**	**Non-recurrent CDI *N* = 35,899 (83.2%)**	**Recurrent CDI *N* = 7,251 (16.8%)**	**Community-acquired *N* = 3,094 (7.2%)**	**Hospital-acquired *N *= 39,526 (91.6)**	**Unknown origin *N* = 530 (1.2%)**	**Control *N* = 355,172**
	N (%)	N (%)	N (%)	N (%)	N (%)	N (%)	N (%)
*Men*	19,780 (45.8)	16,635 (46.3)	3,145 (43.4)	1,209 (39.1)	18,335 (46.4)	236 (44.5)	159,897 (45.0)
*Women*	23,370 (54.2)	19,264 (53.7)	4,106 (56.6)	1,885 (60.9)	21,191 (53.6)	294 (55.5)	195,275 (55.0)
Age at first CDI episode (years)							
< *64*	10,884 (25.2)	9,183 (25.6)	1,701 (23.5)	2,039 (65.9)	8.625 (21.8)	220 (41.5)	107,545 (30.3)
≥ *65*	32,266 (74.8)	26,716 (74.4)	5,550 (76.5)	1,055 (34.1)	30,901 (78.2)	310 (58.5)	247,627 (69.7)
*Median age, years (IQR)*	77 (64–85)	76 (64–85)	77 (66–84)	54 (29–69)	77 (66–85)	68 (51–77)	73 (61–82)
Likely origin							
*Hospital acquired*	39,526 (91.6)	32,962 (91.8)	6,564 (90.5)	-	-	-	-
*Community acquired*	3,094 (7.2)	2,532 (7.1)	562 (7.8)	-	-	-	-
*Unknown*	530 (1.2)	405 (1.1)	125 (1.7)	-	-	-	-
Place of birth							
*Nordic*	40,110 (93.0)	33,335 (92.7)	6,775 (93.4)	36,850 (93.3)	2,768 (89.5)	492 (92.8)	238,711 (67.2)
*Non-Nordic*	2,261 (5.2)	1,907 (5.3)	354 (4.9)	2,023 (5.1)	210 (6.8)	28 (5.3)	19,887 (5.6)
*Missing*	779 (1.8)	657 (1.8)	122 (1.7)	653 (1.7)	116 (3.8)	10 (1.9)	96,574 (27.2)
Charlson comorbidity index score							
*0*	6,126 (14.2)	5,178 (14.4)	948 (13.1)	1,469 (47.5)	4,539 (11.5)	118 (22.3)	144,252 (40.6)
*1*	5,321 (12.3)	4,487 (12.5)	834 (11.5)	564 (18.2)	4,687 (11.9)	70 (13.2)	55,981 (15.8)
*2*	8,090 (18.8)	6,781 (18.9)	1,309 (18.1)	420 (13.6)	7,580 (19.2)	90 (17.0)	62,297 (17.5)
*3*	6,846 (15.9)	5,711 (15.9)	1,135 (15.7)	251 (8.1)	6,501 (16.4)	94 (17.7)	40,283 (11.3)
*4*	5,261 (12.2)	4,340 (12.1)	921 (12.7)	157 (5.1)	5,061 (12.8)	43 (8.1)	22,694 (6.4)
≥ *5*	11,506 (26.7)	9,402 (26.2)	2,104 (29.0)	233 (7.5)	11,158 (28.2)	115 (21.7)	29,665 (8.4)
*Median score (IQR)*	3 (1–5)	3 (1–5)	3 (2–5)	1 (0–2)	3 (2–5)	2 (1–4)	1 (0–3)
Charlson comorbidities							
*Myocardial infarction*	9,559 (22.2)	7,824 (21.8)	1,735 (23.9)	221 (7.1)	9,246 (23.4)	92 (17.4)	44,545 (12.5)
*Congestive heart failure*	834 (1.9)	681 (1.9)	153 (2.1)	19 (0.0)	810 (2.1)	5 (0.9)	2,536 (0.7)
*Peripheral vascular disease*	7,952 (18.4)	6,487 (18.1)	1,465 (20.2)	198 (6.4)	7,666 (19.4)	88 (16.6)	23,065 (6.5)
*Cerebrovascular disease*	12,246 (28.4)	10,137 (28.2)	2,109 (29.1)	363 (11.7)	11,766 (29.8)	117 (22.1)	63,310 (17.8)
*Dementia*	4,393 (10.2)	3,714 (10.4)	679 (9.4)	74 (2.4)	4,282 (10.8)	37 (7.0)	32,580 (9.2)
*Chronic pulmonary disease*	9,945 (23.1)	8,085 (22.5)	1,860 (25.6)	585 (18.9)	9,231 (23.4)	129 (14.3)	39,529 (11.1)
*Connective tissue disease*	4,848 (11.2)	3,904 (10.9)	944 (13.0)	168 (5.4)	4,642 (11.7)	38 (7.2)	16,210 (4.6)
*Peptic ulcer disease*	2,079 (4.8)	1,683 (4.7)	396 (5.5)	104 (3.4)	1,955 (5.0)	20 (3.8)	7,268 (2.1)
*Mild chronic liver disease*	1,809 (4.2)	1,502 (4.2)	307 (4.2)	65 (2.1)	1,722 (4.4)	22 (4.2)	3,992 (1.1)
*Diabetes mellitus without end-organ damage*	10,219 (23.7)	8,529 (23.8)	1,690 (23.3)	296 (9.6)	9,811 (24.8)	112 (21.1)	45,507 (12.8)
*Diabetes mellitus with end-organ damage*	6,749 (15.6)	5,592 (15.6)	1,157 (6.0)	155 (5.0)	6,526 (16.5)	68 (12.8)	22,488 (6.3)
*Hemiplegia or paraplegia*	2,725 (6.3)	2,230 (6.2)	495 (6.8)	65 (2.1)	2,636 (6.7)	24 (4.5)	7,369 (2.1)
*Moderate to severe chronic renal disease*	9,250 (21.4)	7,483 (20.8)	1,767 (24.4)	195 (6.3)	8,970 (22.7)	85 (16.0)	20,886 (5.9)
*Moderate to severe liver disease*	1,064 (2.5)	871 (2.4)	193 (2.7)	23 (0.7)	1,030 (2.6)	11 (2.1)	1,398 (0.4)
*Any malignancy*	18,087 (41.9)	15,051 (41.9)	3,036 (41.9)	709 (22.9)	17,159 (43.4)	219 (41.3)	104,190 (29.3)
*AIDS/HIV*	58 (0.1)	54 (0.2)	4 (0.1)	10 (0.3)	48 (0.1)	0 (0.0)	160 (0.1)
Other comorbidities							
*Hematologic disease*	18,594 (43.1)	15,320 (42.7)	3,274 (45.2)	437 (14.1)	17,963 (45.5)	194 (36.6)	48,702 (13.7)
*Inflammatory bowel disease*	2,059 (4.8)	1,671 (4.7)	388 (5.4)	249 (8.1)	1,746 (4.4)	64 (12.1)	3,172 (0.9)
Prior history of CDI*	502 (1.2)	362 (1.0)	140 (1.9)	42 (1.4)	441 (1.1)	19 (3.6)	-
Total ever-use of prescription drugs							
*Antibiotic ever-use*	41,893 (97.1)	34,748 (96.8)	7,145 (98.6)	3,072 (99.3)	38,291 (96.9)	530 (100.0)	304,851 (85.8)
*Aspirin ever-use*	22,051 (51.1)	18,200 (50.7)	3,851 (53.1)	766 (24.8)	21,057 (53.3)	228 (43.0)	136,404 (38.4)
*Any other prescription drug ever-use*	43,064 (99.8)	35,816 (99.8)	7,248 (100.0)	3,086 (99.7)	39,448 (99.8)	530 (100.0)	355,172 (100.0)
*H2RA ever-use*	3,175 (7.4)	2,546 (7.1)	629 (8.7)	275 (8.9)	2,855 (7.2)	45 (8.5)	16,182 (4.6)
*NSAID ever-use*	25,441 (59.0)	20,954 (58.4)	4,487 (61.9)	2,096 (67.7)	16,530 (41.8)	349 (65.9)	209,911 (59.1)
*PPI ever-use*	30,841 (71.5)	25,441 (70.9)	5,400 (74.5)	1,860 (60.1)	28,582 (72.3)	399 (75.3)	162,218 (45.7)
Median number of entero- and colonoscopies (range)	0 (0–7)	0 (0–7)	0 (0–6)	0 (0–6)	0 (0–7)	0 (0–7)	0 (0–9)

*Abbreviations*: *IQR* Interquartile range, *AIDS* Acquired immunodeficiency syndrome, *HIV* Human immunodeficiency virus, *H2RA* Selective histamine type 2 receptor antagonist, *NSAID* Nonsteroidal anti-inflammatory drug, *PPI* Proton pump inhibitor

^*^Prior history of CDI was defined as having at least one record of CDI diagnosis in the National Patient Registry between 1997 and 2005

Individuals with CDI had a median follow-up time of 2.0 years, compared to 5.5 years for individuals without CDI, 2.1 years for those with rCDI and 1.9 years for those with non-rCDI. Those with community-acquired CDI and hospital-acquired CDI had respectively 5.9 years and 1.7 years of follow-up.

**Fig. 1:**
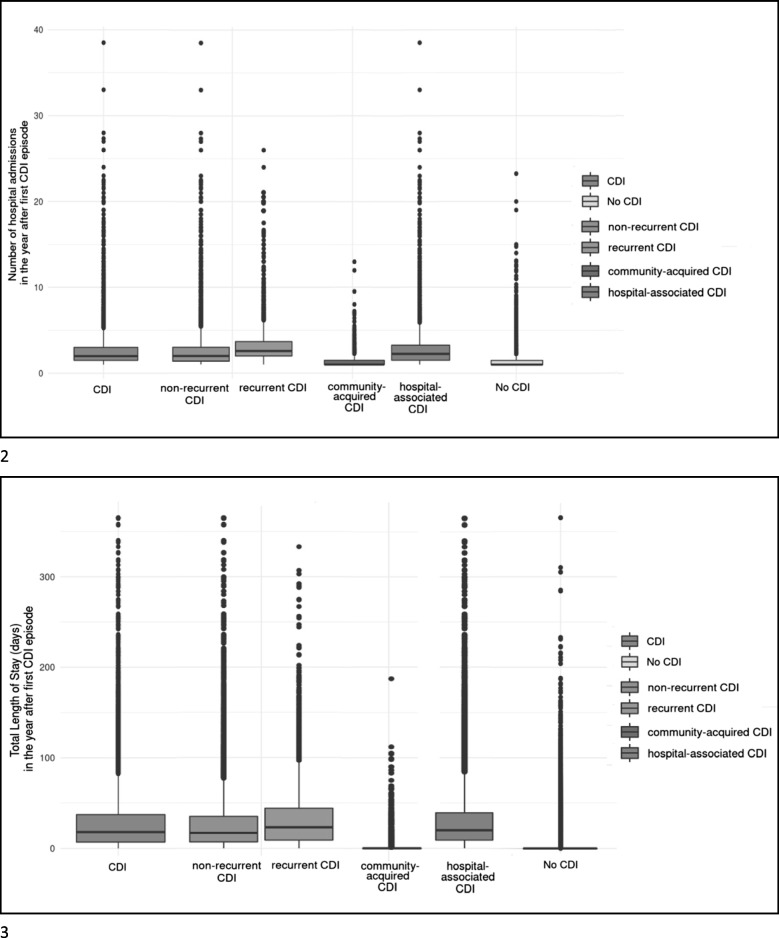
Distribution of health care consumption related to *Clostridioides difficile* infection (CDI), by recurrence status and origin of onset

Individuals with CDI were older, had more comorbidities (higher CCI scores), and more antibiotic, aspirin and PPI prescriptions, compared to those without CDI (Table 1). Differences in age, sex and comorbidities were limited between those with and without recurrence. Individuals with community-acquired CDI were younger than hospital-acquired CDI (65.9% versus 2.18% younger than 65 years), and had less comorbidities (47.5% with CCI score of zero compared to 11.5%) (Table 1). The overall distribution of our 4 outcome parameters is presented in Fig. 1.

### Length of stay

**Fig. 2:**
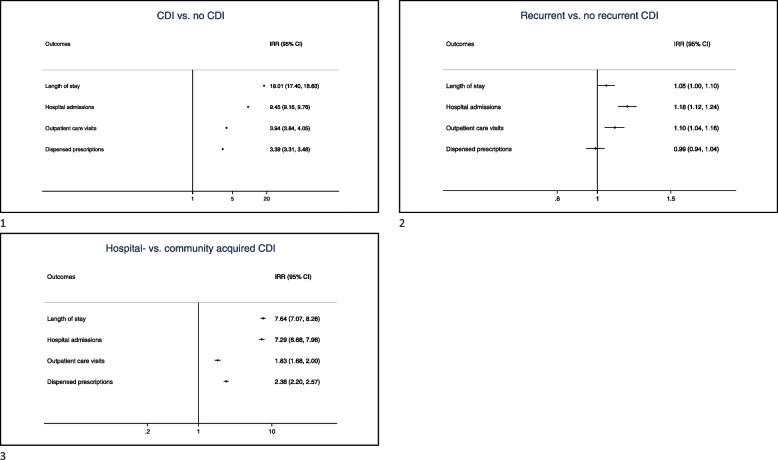
Relative increase of health care consumption expressed as incidence rate ratios (IRR) and 95% confidence intervals (CI), comparing *Clostridioides difficile* infection (CDI) to controls, recurrence to no- recurrence, and hospital-acquired to community-acquired CDI

The average accumulated length of stay within the first year after the first CDI episode was 27.4 days for individuals with CDI and 1.6 days for individuals without CDI (Table 2), resulting in IRR = 18.01 for the entire follow-up period (95% CI 17.40–18.63)(Fig. 2). Those with recurrence stayed on average an accumulated 31.9 days, versus 26.5 days in those without recurrence during the first year since the initial CDI (IRR = 1.05, 95% CI 1.00–1.10)(Table 2, Fig. 2). Individuals with hospital-acquired CDI stayed on average 29.5 days, versus 2.9 days in those with community-acquired CDI (IRR = 7.64, 95% CI 7.07–8.26) (Table 2, Fig. 2). Results were similar for the entire follow-up period. (Additional Table 3).

**Table 2:** Summary statistics for healthcare consumption within one year from the first *Clostridioides difficile* infection (CDI) or its proxy (controls), stratified by recurrence

	**CDI (***N* = 43,150)		**Non-recurrent CDI (***N* = 35,899)		Recurrent CDI*(*N* = 7,251)		**Hospital-acquired CDI** (*N* = 39,526)		**Community acquired CDI** (*N* = 3,094)		**Control (***N* = 355,172)	
	Mean (SD)	Median (IQR)	Mean (SD)	Median (IQR)	Mean (SD)	Median (IQR)	Mean (SD)	Median (IQR)	Mean (SD)	Median (IQR)	Mean (SD)	Median (IQR)
**Length of stay, in days**												
*Total*	27.4 (30.8)	18.0 (7.0–37.0)	26.5 (30.3)	17.0 (7.0–35.0)	31.9 (32.9)	23.0 (9.0–44.0)	29.5 (31.1)	20.0 (9.0–39.0)	2.9 (9.9)	0.0 (0.0–0.0)	1.6 (7.2)	0.0 (0.0–0.0)
*Hospital acquired*	29.5 (31.1)	20.0 (9.0–39.0)	28.5 (30.6)	19.0 (9.0–37.0)	34.5 (33.1)	25.0 (12.0–47.0)	-	-	-	-	-	-
*Community acquired*	2.9 (9.9)	0.0 (0.0–0.0)	2.4 (9.2)	0.0 (0.0–0.0)	5.2 (12.1)	0.0 (0.0–5.0)	-	-	-	-	-	-
**Number of in-hospital admissions**												
*Total*	2.6 (1.8)	2.0 (1.5–3.0)	2.5 (1.7)	2.0 (1.4–3.0)	3.0 (1.9)	2.6 (2.0–3.7)	2.7 (1.8)	2.3 (1.5–3.3)	1.4 (0.9)	1.0 (1.0–1.5)	1.3 (0.7)	1.0 (1.0–1.5)
*Hospital acquired*	2.7 (1.8)	2.3 (1.5–3.3)	2.6 (1.7)	2.0 (1.5–3.0)	3.2 (2.0)	2.7 (2.0–3.8)	-	-	-	-	-	-
*Community acquired*	1.4 (0.9)	1.0 (1.0–1.5)	1.4 (0.9)	1.0 (1.0–1.5)	1.5 (0.8)	1.0 (1.0–1.8)	-	-	-	-	-	-
**Number of outpatient care visits**												
*Total*	4.0 (10.2)	2.0 (1.0–3.4)	3.9 (10.0)	2.0 (1.0–3.3)	4.5 (11.3)	2.1 (1.3–3.8)	4.1 (10.5)	2.0 (1.0–3.5)	2.7 (5.2)	2.0 (1.0–2.8)	1.9 (2.6)	1.3 (1.0–2.0)
*Hospital acquired*	4.1 (10.5)	2.0 (1.0–3.5)	4.0 (10.3)	2.0 (1.0–3.4)	4.5 (11.6)	2.1 (1.3–3.7)	-	-	-	-	-	-
*Community acquired*	2.7 (5.2)	2.0 (1.0–2.8)	2.6 (5.0)	1.8 (1.0–2.6)	3.4 (6.1)	2.3 (1.8–3.8)	-	-	-	-	-	-
**Number of dispensed prescriptions**												
*Total*	25.5 (30.5)	14.8 (7.7–30)	25.2 (30.4)	14.6 (7.5–29.8)	26.8 (21.4)	16.0 (8.6–31.4)	26.6 (31.0)	15.7 (8.3–31.5)	13.0 (19.8)	6.8 (3.0–14.4)	13.7 (21.1)	6.7 (2.8–14.7)
*Hospital acquired*	26.6 (31.0)	15.7 (8.3–31.5)	26.3 (30.9)	15.4 (8.1–31.2)	28.0 (31.9)	16.9 (9.2–33.0)	-	-	-	-	-	-
*Community acquired*	13.0 (19.8)	6.8 (3–14.4)	21.6 (19.5)	6.5 (3.0–14.0)	14.7 (21.1)	7.8 (3.6–16.7)	-	-	-	-	-	-

*Abbreviations*: *Non-rCDI* Non-recurrent CDI episode, *rCDI* Recurrent CDI episode, *SD* Standard deviation, *IQR* Interquartile range. *rCDI Recurrent CDI was defined as having a recorded CDI episode within eight weeks from the start of the last CDI episode

### In-hospital admissions

Within the first year after the first CDI episode, the average number of in-hospital admissions was 2.6 for individuals with CDI and 1.3 for individuals without CDI (Table 2), a nine times higher number of admissions after adjustments for the entire study period (IRR = 9.45; 95% CI 9.16–9.76)(Fig. 2). Those with recurrence had on average 3.0 admissions, versus 2.5 in those without recurrence (Table 2), resulting in IRR = 1.18 (95% CI 1.12–1.24)(Fig. 2). Hospital-acquired CDI resulted in 2.7 admissions versus 1.4 admissions in community-acquired CDI (Table 2), with IRR = 7.29 (95% CI 6.68–7.96) (Fig. 2).

### Outpatient visits

Individuals with CDI had an average of 4.0 outpatient visits, compared to 1.9 among controls (Table 2), resulting in IRR = 3.94 for the entire study period (95% CI 3.84–4.05) (Fig. 2). Those with recurrence had on average 4.5 outpatient care visits, compared to 3.9 in those without recurrence (IRR = 1.10, 95% CI 1.04–1.16) (Table 2, Fig. 2). Individuals with hospital-acquired CDI had on average 4.1 outpatient care visits compared to 2.7 among those with community-acquired CDI (IRR = 1.83, 95% CI 1.68–2.00)(Table 2, Fig. 2).

### Prescription drugs

On average, those with CDI dispensed 25.5 prescriptions, compared to 13.7 among controls (IRR = 2.38, 95% CI 2.20–2.57) (Table 2, Fig. 2). Those with recurrence dispensed on average 26.8 prescriptions, compared to 25.2 in those without recurrence (IRR = 0.99, 95% CI 0.94–1.04) (Table 2, Fig. 2). Individuals with hospital-acquired CDI dispensed 26.6 prescriptions compared to 13.0 among those with community-acquired CDI (IRR = 2.38, 95% 2.20–2.57) (Table 2, Fig. 2).

### Covariates

**Fig. 3:**
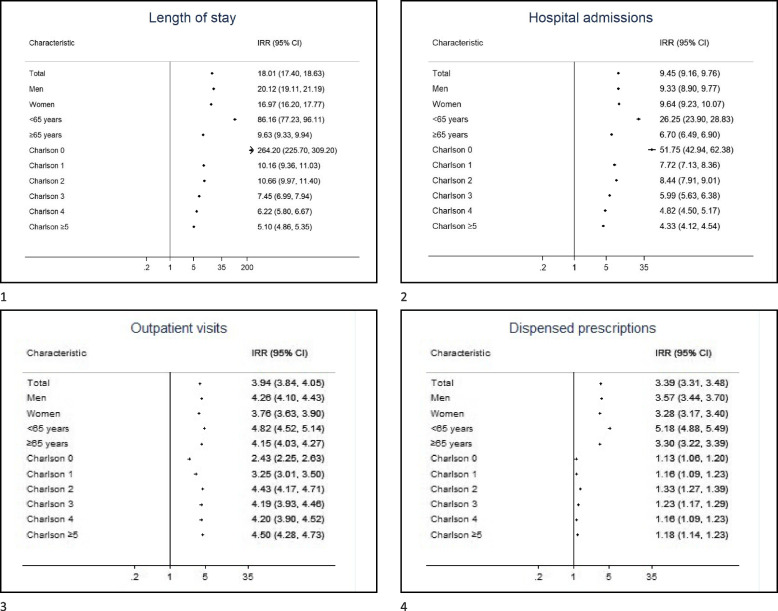
Impact of sex, age and comorbidities on the relative increase in healthcare consumption related to Clostridioides difficile infection (CDI), expressed as incidence rate ratios (IRR) and 95% confidence intervals (CI)

Compared to their respective controls without CDI, all healthcare resource measures were higher among all CDI subgroups by sex, age and comorbidity (Fig. 3). The IRR for length of stay, outpatient care visits and dispensed prescriptions were slightly higher in men than women, while number of hospital admissions was similar (Fig. 3). All IRR were higher for the youngest age group (< 65 years) than for the older (≥ 65 years), particularly for length of stay (IRR = 86.16, 95% CI 77.23–96.11 compared to IRR = 9.63, 95% CI 9.33–9.94) and hospital admissions (IRR = 26.25, 95% CI 23.90–28.83 compared to IRR = 6.70, 95% CI 6.49–6.90) (Fig. 3). The IRR for length of stay and number of hospital admissions among those without comorbidities was much higher than in those with comorbidities with respectively IRR = 264.20 (95%CI 225.70–309.20) and IRR = 51.75 (95% CI 42.94–62.38) for CCI score zero, compared to IRR = 5.10 (95% CI 4.86–5.35) and IRR = 4.33 (95% CI 4.12–4.54) for CCI score ≥ 5 (Fig. 3). The IRR for outpatient visits seemed to increase with increasing number of comorbidities; while the IRR for dispensed prescriptions did not change much for different comorbidity groups (Fig. 3). More detailed descriptive statistics by age, sex and comorbidities are shown in Additional Table 3 for recurrence status, and Additional Table 4 for hospital-acquired versus community acquired CDI within one year after the infection; and in Additional Tables 5–6 the total study period.

#### Absolute burden of CDI

**Fig. 4:**
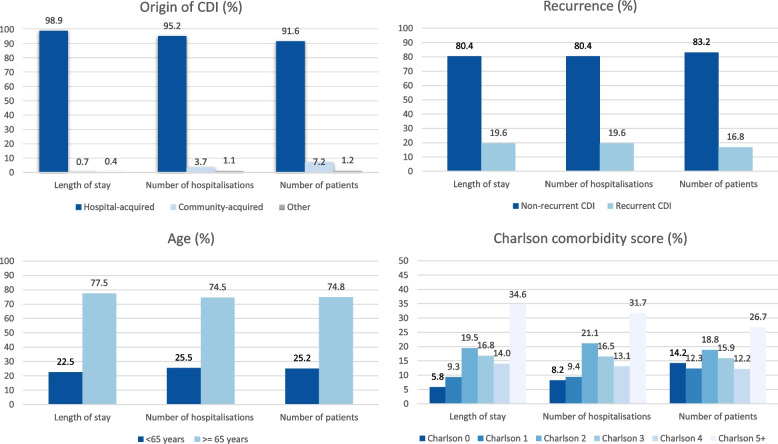
Distribution of cases within the total group with *Clostridioides difficile* infection (CDI) by different characteristics: which subgroups are largest (presented as %)?

Overall, there were 1,177,761 hospitalisation days; and 111,494 hospitalisations during the first year after CDI, in the entire CDI group (43,150 individuals)(Additional Table 7). The 74.8% of the population older than 65 years contributed to 77.5% of overall share of hospitalisation days and 74.5% of all admissions during the first year after CDI (Additional Table 7, Fig. 4). The 91.6% of the CDI group with hospital-acquired CDI, contributed to almost all hospitalisation days (98.9%) and the large majority of hospitalisations (95.2%)(Additional Table 7, Fig. 4). The 16.8% of individuals with CDI presenting with at least one recurrence, contributed 19.6% of both hospitalisation days and hospitalisations (Additional Table 7, Fig. 4). Those individuals without comorbidities (14.2% of the CDI cohort), contributed with only 5.8% and 8.2% of the hospitalisation days and hospitalisations respectively; while the share of hospitalisation days and hospitalisations was larger for all with at least two comorbidities (Additional Table 7, Fig. 4).

## Discussion

This study is probably the largest nationwide and population-based European cohort following individuals with CDI with the aim to estimate CDI-associated healthcare consumption by comparing the onset of CDI and recurrence status among different age and comorbidity groups. The results show that individuals with CDI consume substantially more healthcare resources than those without. Individuals with CDI had, on average, an 18-fold longer length of stay, ninefold higher rate of in-hospital admissions, fourfold higher rate of outpatient visits and threefold higher rate of dispensed prescriptions than their individually matched controls without CDI after adjustments for comorbidities and other confounders. Those with recurrence had 18% more hospital admissions, and 10% more outpatient care visits than those without recurrence, with limited differences in accumulated length of stay, and number of dispensed prescriptions. Hospital-acquired CDI resulted in 7–8 times the rate of hospital admissions and a higher length of stay than among those with community-acquired CDI, with approximately twofold higher rate of outpatient care visits and dispensed prescriptions. From the previous systematic reviews, one can easily depict how this translates into substantial healthcare costs, and combined with the relatively high incidence, would be resulting in a substantial pressure on the healthcare organisations and budgets [2, 9, 10].

Most of our population with CDI was older than 65 years of age (75%) and had a higher number of chronic comorbidities (86%), resulting in the largest overall healthcare burden related to CDI. Nevertheless, the largest *relative* increases in healthcare consumption were seen among those younger than 65 years and those without comorbidities, particularly for the accumulated length of stay and number of hospital admissions, indicating that they clearly have higher healthcare needs than their “healthy” peers. Younger people are generally less frail and require less healthcare. Therefore, a slight increase in the number of admissions, visits, and prescriptions might still be impactful overall, also among the younger patients. For older individuals, healthcare consumption may be more impacted by underlying chronic comorbidities, which may worsen as a result of a CDI. Yet, we did adjust all results for the CCI comorbidity score, and explored the effect of comorbidity on our outcomes as well. Unfortunately, we could not explore the effect of further progression or deterioration of the underlying disease (over time or in relation to the CDI infection), but we acknowledge the health effects of many chronic diseases may change over time (e.g. exacerbations of chronic pulmonary disease or flare ups of inflammatory bowel disease). We also acknowledge that different underlying diseases may result in different risks of CDI, rCDI, and healthcare expenditure patterns.

Our findings do confirm previous findings that recurrence does come with an increase in healthcare consumption compared to those without recurrence, [10, 26, 27] particularly in the number of hospital admissions. Although our study did not find major differences in age, sex and comorbidities between individuals with and without recurrence, age and comorbidities have been identified as important prognostic factors in previous systematic reviews [2, 28]. The differences between healthcare-acquired and community-acquired CDI were, however more pronounced, with clearly more and lengthier hospital admissions among the hospital-acquired CDI group, while the costs of community-acquired CDI are still markedly higher than among controls, as also seen in previous studies [18, 29]. We do point out that there may be some differential misclassification of community-acquired CDI as hospital-acquired, as we classified all with a diagnosis during (or within 4 weeks after) in-hospital admission as hospital-acquired CDI, as the exact diagnoses dates are not recorded in the patient registry. Misclassification as community-acquired CDI was unlikely, and unclear cases were classified as “other.”

The population-based individually-matched design, long follow-up, high-quality data sources, and ability to stratify for chronic comorbidities are the main strengths of our study. Our study had a follow-up period of 14 years, and was not restricted to only inpatients or elderly as in several previous studies [13, 26, 27]. The Patient Registry captures approximately 85–95% of inpatient care diagnoses (positive predictive value), [30] and 80% of all hospital-based outpatient healthcare [30, 31]. We did not find any validation studies specifically assessing the CDI coding in the Swedish Patient Registry although suspect CDI may still be underreported. We do expect the most severe cases are identified, while less severe cases may not be recognised and/or tested as CDI, and not be recorded in the Patient Registry. There is no mandatory reporting of CDI (not on the list of notifiable infections). At least 84 controls (0.02%) were misclassified as not having CDI while CDI was reported as their main or underlying cause of death. Nevertheless, the reporting of CDI is likely more complete in Sweden than many other countries, as suggested by the relatively high reported CDI incidence in Sweden [2, 32, 33]. The Swedish Prescribed Drug Registry captures 45–100% of the entire population annually (depending on age group), [34] about 70% of the population in 2019, [34] and 85% between 2005 and 2014, [35] with overall less than 0.3% missing patient identify data [23]. We therefore consider the drug registry as sufficiently representative of the Swedish population for selecting the controls. The Causes of Death Register captures approximately 98–99% of all causes of death [36].

However, we did not have information on specific CDI strains, as some are associated with more severe disease (higher risk of toxic megacolon and colon perforation) and consequently higher healthcare cost [37]. We also lack information on body mass index and frailty which are not collected in the nationwide Swedish registries, yet may be important and under-investigated predictors of healthcare consumption, particularly among the elderly. We also focussed on four main healthcare consumption parameters (hospitalised days, admissions, outpatient care visits and prescriptions), while other parameters also add to the costs, including colonoscopies, imaging, surgery, surgical complications, parental nutrition, blood transfusions, and type of ward (intensive care unit or not) [10, 38]. Furthermore, the presented results cover all healthcare services among different patient populations, and may not necessarily be directly linked to CDI.

Our findings support the importance of prevention measures for CDI and especially CDI recurrence for individual patients as well as at population level, which includes rational use of antimicrobials and other drugs including PPIs [39, 40]. Yet, the health economic impact of different treatment options and combinations also needs further investigation [41–43].

## Conclusions

To conclude, CDI, as well as rCDI, were associated with increased health consumption, in both sexes and all age and comorbidity groups, particularly regarding the length of stay and number of hospitalisations. Our findings confirm that CDI results in an important and substantial incremental healthcare burden, in absolute numbers especially among the elderly, yet relatively speaking especially among the younger compared to their healthy peers.

### Supplementary Information

**Additional file 1: Additional Table 1. **International Classification of Disease (ICD)-10 codes applied for calculation of the Charlson Comorbidity Index score ascertained from the national Patient Registry (in- and outpatient care). **Additional Table 2.** The prescribed drug groups considered in this study, as retrieved from the Swedish Prescribed Drug Registry. **Additional Table 3.** Recurrence of Clostridioides difficile infection (CDI): Summary statistics for healthcare consumption within one year from the first infection or its proxy (controls). **Additional Table 4.** Origin of Clostridioides difficile infection (CDI): Summary statistics for healthcare consumption within one year from the first infection or its proxy (controls). **Additional Table 5.** Clostridioides difficile infection (CDI) and recurrence: descriptive statistics for total length of in-hospital stay (LOS) and total number of hospital admissions, stratified by sex, age, Charlson comorbidity index score, and likely origin (Total study period 2006- 2019). **Additional Table 6.** Origin of the Clostridioides difficile infection (CDI): Descriptive statistics for total length of in-hospital stay (LOS) and total number of hospital admissions, stratified by sex, age, Charlson comorbidity index score (Total study period 2006-2019). **Additional Table 7.** Total burden of Clostridioides difficile infection (CDI) in terms of number of patients, length of stay and number of hospitalisations during the first year and during the entire study period. 

## Data Availability

The datasets generated and analysed during the current study are not publicly available due to restrictions from the National Board of Health and Welfare (Socialstyrelsen), who own the data. The data are available from the corresponding author (NB) on reasonable request after required approvals from the national Ethics Committee and National Board of Health and Welfare are obtained.

## Abbreviations

CCI: Charlson comorbidity index
CDI: *Clostridioides difficile* Infection
CI: Confidence interval
IRR: Incidence rate ratio
LOS: Length of stay
USD: United States Dollars
