# Supplementary material for: Clostridioides difficile infection, recurrence and the associated healthcare consumption in Sweden between 2006 and 2019: a population-based cohort study
Source: BMC Infect Dis. 2024 May 3;24:468. doi: 10.1186/s12879-024-09364-3 (PMC11067081; doi:10.1186/s12879-024-09364-3)
Supplement: Supplementary file 1 — Additional file 1: Additional Table 1. International Classification of Disease (ICD)-10 codes applied for calculation of the Charlson Comorbidity Index score ascertained from the national Patient Registry (in- and outpatient care). Additional Table 2. The prescribed drug groups considered in this study, as retrieved from the Swedish Prescribed Drug Registry. Additional Table 3. Recurrence of Clostridioides difficile infection (CDI): Summary statistics for healthcare consumption within one year from the first infection or its proxy (controls). Additional Table 4. Origin of Clostridioides difficile infection (CDI): Summary statistics for healthcare consumption within one year from the first infection or its proxy (controls). Additional Table 5. Clostridioides difficile infection (CDI) and recurrence: descriptive statistics for total length of in-hospital stay (LOS) and total number of hospital admissions, stratified by sex, age, Charlson comorbidity index score, and likely origin (Total study period 2006- 2019). Additional Table 6. Origin of the Clostridioides difficile infection (CDI): Descriptive statistics for total length of in-hospital stay (LOS) and total number of hospital admissions, stratified by sex, age, Charlson comorbidity index score (Total study period 2006-2019). Additional Table 7. Total burden of Clostridioides difficile infection (CDI) in terms of number of patients, length of stay and number of hospitalisations during the first year and during the entire study period. [file 12879_2024_9364_MOESM1_ESM.docx]

**Additional Files**

- **Additional Table 1**: International Classification of Disease (ICD)-10 codes applied for calculation of the Charlson Comorbidity Index score ascertained from the national Patient Registry (in- and outpatient care).
- **Additional Table 2**: The prescribed drug groups considered in this study, as retrieved from the Swedish Prescribed Drug Registry.
- **Additional Table 3:** Recurrence of Clostridioides difficile infection (CDI): Summary statistics for healthcare consumption within one year from the first infection or its proxy (controls)
- **Additional Table 4:** Origin of *Clostridioides difficile* infection (CDI): Summary statistics for healthcare consumption within one year from the first infection or its proxy (controls).
- **Additional Table 5:** *Clostridioides difficile* infection (CDI) and recurrence: descriptive statistics for total length of in-hospital stay (LOS) and total number of hospital admissions, stratified by sex, age, Charlson comorbidity index score, and likely origin (Total study period 2006- 2019).
- **Additional Table 6:** Origin of the *Clostridioides difficile* infection (CDI): Descriptive statistics for total length of in-hospital stay (LOS) and total number of hospital admissions, stratified by sex, age, Charlson comorbidity index score (Total study period 2006-2019).
- **Additional Table 7**: Total burden of *Clostridioides difficile* infection (CDI) in terms of number of patients, length of stay and number of hospitalisations during the first year and during the entire study period

**Additional Table 1: International Classification of Disease (ICD) codes applied for selected diseases and calculation of the Charlson Comorbidity Index score, ascertained from the national Patient Registry (in- and outpatient care).**

| **Comorbidity** | **Charlson Comorbidity index weight** | **Corresponding ICD-10 codes** |
| --- | --- | --- |
| Myocardial infarction | 1 | I21-I23, I241, I252 |
| Congestive heart failure | 1 | I110, I130, I132-255, I420, I425-I439, I50, K761 |
| Peripheral vascular disease | 1 | A520, I70-I72, I731, I738, I739, I771, I778, I790, K441 |
| Cerebrovascular disease | 1 | G45-G46, I60–I69, H340 |
| Dementia | 1 | F00–F03, F051, G30, G311 |
| Chronic pulmonary disease | 1 | I270, I278, I279, J40-J47, J60-J67, J684, J701, J703 |
| Connective tissue disease | 1 | M05-M06, M08-09, M315, M32–M34, M351, M353 |
| Ulcer | 1 | K25-K28 |
| Mild (chronic) liver disease | 1 | B18, K700-K704, K709, K710, K713-K719, K73-K74, K760 |
|  |  |  |
| Diabetes mellitus without end-organ damage | 1 | E109, E119 |
| Any malignancy*, including metastatic cancer, leukaemia, and lymphoma | 2 | C00-C97, ** omitting non-melanoma skin cancer C43.0-9 and C87(which is not used in Sweden)* |
| Diabetes mellitus with end-organ damage | 2 | E129, E139, E149, E100-E108, E110-E118, E120-E128, E130-E138, E140-E148 |
| Hemiplegia or paraplegia | 2 | G041, G114, G801, G802, G810-G82 |
| Moderate to severe chronic renal disease | 2 | I120, I131, I132, N03-N05, N18-N19, N25-N26, Z49 |
| Moderate or severe liver disease | 3 | I810-I820, I85, I864, K704, K711, K712, K72, K762, K763, K765-K767 |
| AIDS/HIV | 6 | B200–B248 |
| Inflammatory bowel disease | - | K500-K501, K508, K510, K512, K515, K518, K513, K523 |
| Hematologic disease | - | D50-D89 |

Abbreviations: AIDS/HIV: acquired immunodeficiency syndrome/ human immunodeficiency virus.

**Additional Table 2: The prescribed drug groups considered in this study, as retrieved from the Swedish Prescribed Drug Registry.**

| **Main drug group** | **Specification** | **ATC codes** |
| --- | --- | --- |
| Systemic antibiotics | Antibacterial for systemic use | J01 |
|  | Nitroimidazole derivatives | P01, P01A1 |
| Gastric acid regulating drugs | Drugs for peptic ulcer and gastro-esophageal reflux  H2-receptor antagonists  Proton pump inhibitors | A02B  A02BA  A02BC |
| Drugs used in diabetes | Including blood glucose lowering drugs, and insulins and analogues | A10 |
| Drugs for constipation | Including osmotic laxatives | A06 |
| Anti-diarrheal, intestinal anti-inflammatory-anti-infective agents | Including intestinal anti-inflammatory agents, incl. mesalamine | A07 |
| Beta-blockers | Beta-blocking agents | C07 |
| Lipid-modifying agents | Including statins etc. | C10 |
| Corticosteroids for systemic use |  | H02 |
| Nervous system | Opioids | N02A |
|  | Anti-epileptics (including benzodiazepines) | N03, N03AE01 |
|  | Psycholeptics (including benzodiazepines and benzodiazepine-like drugs | N05, N05BA, N05CD, N05CF |
| Anti-inflammatory drugs | Selective Cox-2 inhibitors and other non-selective COX inhibitors | M01AH |
|  | Aspirin | B01AC06, N02BA |
| All drugs | All outpatient care dispensed drugs | All ATC codes |

Abbreviations: ATC: Anatomic Therapeutic Classification code.

**Additional Table 3: Recurrence of *Clostridioides difficile* infection (CDI): Summary statistics for healthcare consumption within one year from the first infection or its proxy (controls)**

|  | **CDI**  **(**N=43,150) | | **Non-recurrent CDI (**N=35,899) | | **Recurrent CDI* (**N=7,251) | | **Control**  **(**N=355,172) | |
| --- | --- | --- | --- | --- | --- | --- | --- | --- |
|  | Mean (SD) | Median (IQR) | Mean (SD) | Median (IQR) | Mean (SD) | Median (IQR) | Mean (SD) | Median (IQR) |
| **Length of stay, in days** |  |  |  |  |  |  |  |  |
| *Total* | 27.4 (30.8) | 18.0 (7.0-37.0) | 26.5 (30.3) | 17.0 (7.0-35.0) | 31.9 (32.9) | 23.0 (9.0-44.0) | 1.6 (7.2) | 0.0 (0.0-0.0) |
| *Men* | 28.9 (31.9) | 20.0 (8.0-39.0) | 28.0 (31.3) | 19.0 (8.0-37.0) | 33.9 (34.7) | 24.0 (11.0-46.0) | 1.7 (7.6) | 0.0 (0.0-0.0) |
| *Women* | 26.1 (29.8) | 17.0 (6.0-35.0) | 25.2 (29.3) | 16.0 (6.0-34.0) | 30.4 (31.3) | 21.0 (8.0-42.0) | 1.5 (6.8) | 0.0 (0.0-0.0) |
| *≤64 years* | 24.5 (39.4) | 8.0 (2.0-31.0) | 24.2 (39.3) | 8.0 (2.0-30.0) | 26.1 (40.3) | 9.0 (3.0-33.0) | 0.3 (3.7) | 0.0 (0.0-0.0) |
| *≥65 years* | 28.4 (27.3) | 21.0 (10.0-38.0) | 27.2 (26.5) | 20.0 (9.0-36.0) | 33.7 (30.0) | 25.0 (13.0-46.0) | 2.1 (8.2) | 0.0 (0.0-0.0) |
| *By Charlson comorbidity index scores:* |  |  |  |  |  |  |  |  |
| *0* | 11.2 (18.3) | 5.0 (1.0-14.0) | 11.0 (18.0) | 4.0 (1.0-14.0) | 12.5 (19.5) | 5.0 (2.0-16.0) | 0.1 (1.8) | 0.0 (0.0-0.0) |
| *1* | 20.8 (25.6) | 13.0 (5.0-28.0) | 20.1 (24.5) | 13.0 (4.0-27.0) | 24.4 (30.2) | 16.0 (6.0-32.0) | 1.3 (5.8) | 0.0 (0.0-0.0) |
| *2* | 28.5 (32.0) | 19.0 (8.0-37.0) | 28.0 (31.6) | 19.0 (8.0-36.0) | 31.2 (33.5) | 21.0 (9.0-41.0) | 1.8 (7.5) | 0.0 (0.0-0.0) |
| *3* | 28.9 (30.0) | 20.0 (9.0-38.0) | 27.8 (29.1) | 19.0 (9.0-37.0) | 34.1 (33.5) | 25.0 (11.0-47.0) | 2.7 (9.0) | 0.0 (0.0-0.0) |
| *4* | 31.5 (31.3) | 23.0 (11.0-42.0) | 30.6 (31.1) | 22.0 (10.0-41.0) | 35.5 (31.7) | 27.0 (14.0-49.0) | 3.8 (11.2) | 0.0 (0.0-2.0) |
| *≥5* | 35.4 (33.8) | 26.0 (13.0-47.0) | 34.1 (33.6) | 24.0 (12.0-45.0) | 41.3 (34.2) | 32.0 (17.0-57.0) | 5.4 (13.3) | 0.0 (0.0-5.0) |
| **Number of in-hospital admissions** | |  |  |  |  |  |  |  |
| *Total* | 2.6 (1.8) | 2.0 (1.5-3) | 2.5 (1.7) | 2.0 (1.4-3.0) | 3.0 (1.9) | 2.6 (2.0-3.7) | 1.3 (0.7) | 1.0 (1.0-1.5) |
| *Men* | 2.7 (1.8) | 2.3 (1.5-3.3) | 2.6 (1.8) | 2.2 (1.5-3.1) | 3.2 (2.1) | 2.8 (2.0-4.0) | 1.4 (0.7) | 1.0 (1.0-1.5) |
| *Women* | 2.5 (1.7) | 2.0 (1.3-3.0) | 2.4 (1.7) | 2.0 (1.7-3.0) | 2.9 (1.8) | 2.5 (1.8-3.5) | 1.4 (0.6) | 1.0 (1.0-1.4) |
| *≤64 years* | 2.6 (2.5) | 2.0 (1.0-3.1) | 2.6 (2.4) | 1.9 (1.0-3.0) | 3.0 (2.8) | 2 (1.0-3.5) | 1.1 (0.5) | 1.0 (1.0-1.0) |
| *≥65 years* | 2.6 (1.5) | 2.2 (1.6-3.0) | 2.5 (1.4) | 2.0 (1.4-3.0) | 3.0 (1.6) | 2.7 (2.0-3.7) | 1.4 (0.7) | 1.0 (1.0-1.7) |
| *By Charlson comorbidity index scores:* |  |  |  |  |  |  |  |  |
| *0* | 1.5 (0.9) | 2.0 (1.0-2.0) | 1.4 (0.8) | 1.0 (1.0-1.7) | 1.9 (1.2) | 1.6 (1.0-2.0) | 1.0 (0.2) | 1.0 (1.0-1.0) |
| *1* | 2.0 (1.2) | 1.7 (1.0-2.5) | 1.9 (1.1) | 1.6 (1.0-2.3) | 2.4 (1.6) | 2.0 (1.5-3.0) | 1.3 (0.6) | 1.0 (1.0-1.5) |
| *2* | 2.9 (2.3) | 2.3 (1.5-3.5) | 2.8 (2.3) | 2.0 (1.5-3.4) | 3.3 (2.5) | 1.0 (1.0-1.7) | 1.5 (0.8) | 1.0 (1.0-1.7) |
| *3* | 2.7 (1.8) | 2.2 (1.6-3.2) | 2.6 (1.7) | 2.0 (1.5-3.0) | 3.1 (1.9) | 2.6 (2.0-3.7) | 1.6 (0.8) | 1.3 (1.0-2.0) |
| *4* | 2.8 (1.7) | 2.3 (1.8-3.3) | 2.7 (1.6) | 2.3 (1.7-3.2) | 3.2 (1.9) | 2.8 (2.0-3.8) | 1.8 (0.9) | 2.0 (1.5-2.0) |
| *≥5* | 3.1 (1.6) | 2.7 (2.0-3.7) | 3.0 (1.6) | 2.6 (2.0-3.6) | 3.4 (1.7) | 3.0 (2.3-4.0) | 1.9 (0.9) | 1.8 (1.3-2.3) |
| **Number of outpatient care visits** | |  |  |  |  |  |  |  |
| *Total)* | 4.0 (10.2) | 2.0 (1.0-3.4) | 3.9 (10.0) | 2.0 (1.0-3.3) | 4.5 (11.3) | 2.1 (1.3-3.8) | 1.9 (2.6) | 1.3 (1.0-2.0) |
| *Men* | 4.7 (12.3) | 2.0 (1.0-3.8) | 4.5 (11.8) | 2.0 (1.0-3.7) | 5.6 (14.7) | 2.3 (1.5-4.0) | 2.0 (3.1) | 1.5 (1.0-2.0) |
| *Women* | 3.4 (7.9) | 2.0 (1.0-3.1) | 3.4 (8.0) | 1.8 (1.0-3.0) | 3.6 (7.6) | 2.0 (1.3-3.5) | 1.8 (2.2) | 1.3 (1.0-2.0) |
| *≤64 years* | 5.2 (11.2) | 2.3 (1.3-2.0) | 5.1 (11.3) | 2.2 (1.3-4.7) | 5.5 (10.7) | 2.6 (1.7-5.3) | 1.9 (2.4) | 1.1 (1.0-2.0) |
| *≥65 years* | 3.6 (9.8) | 2.0 (1.0-3.0) | 3.5 (9.4) | 1.9 (1.0-3.0) | 4.2 (11.5) | 2.0 (1.3-3.4) | 1.9 (2.7) | 1.3 (1.0-2.0) |
| *By Charlson comorbidity index scores:* |  |  |  |  |  |  |  |  |
| *0* | 1.8 (1.8) | 1.0 (1.0-2.0) | 1.7 (1.7) | 1.0 (1.0-2.0) | 2.3 (2.1) | 1.8 (1.0-2.7) | 1.3 (0.7) | 1.0 (1.0-1.4) |
| *1* | 1.8 (1.5) | 1.3 (1.0-2.0) | 1.7 (1.4) | 1.1 (1.0-2.0) | 2.1 (1.8) | 1.5 (1.0-2.5) | 1.4 (0.8) | 1.0 (1.0-1.5) |
| *2* | 4.1 (7.0) | 2.0 (1.0-4.5) | 4.1 (6.9) | 2.0 (1.0-4.5) | 4.3 (7.1) | 2.2 (1.4-4.3) | 2.3 (2.7) | 1.5 (1.0-2.3) |
| *3* | 3.6 (7.8) | 2.0 (1.3-3.5) | 3.6 (7.9) | 2.0 (1.1-3.5) | 3.8 (7.2) | 2.1 (1.5-3.7) | 2.2 (2.5) | 1.7 (1.0-2.3) |
| *4* | 4.0 (9.8) | 2.0 (1.3-3.5) | 3.9 (9.6) | 2.0 (1.3-3.5) | 4.3 (10.3) | 2.2 (1.4-3.6) | 2.2 (3.5) | 1.7 (1.2-2.8) |
| *≥5* | 6.3 (16.1) | 2.6 (1.7-4.4) | 6.1 (15.7) | 2.6 (1.7-4.3) | 6.8 (17.7) | 2.7 (1.8-4.5) | 2.6 (5.1) | 2.0 (1.4-2.7) |
| **Number of dispensed prescriptions** | |  |  |  |  |  |  |  |
| *Total* | 25.5 (30.5) | 14.8 (7.7-30.0) | 25.2 (30.4) | 14.6 (7.5-29.8) | 26.8 (21.4) | 16.0 (8.6-31.4) | 13.7 (21.1) | 6.7 (2.8-14.7) |
| *Men* | 22.6 (27.3) | 13.6 (7.3-25.9) | 22.4 (27.1) | 13.3 (7.1-25.5) | 24.0 (28.5) | 14.9 (8.3-27.3) | 11.8 (18.2) | 6.3 (2.6-12.9) |
| *Women* | 27.9 (32.8) | 16.3 (8.1-34.0) | 27.7 (32.2) | 16.1 (8-33.8) | 29.0 (33.2) | 17.1 (8.9-35.3) | 15.3 (23.1) | 7.1 (2.9-16.6) |
| *≤64 years* | 19.5 (30.9) | 9.1 (4.2-20.3) | 19.2 (30.5) | 9.0 (4.1-20.1) | 20.9 (33.4) | 10.0 (4.5-21.8) | 5.5 (11.5) | 2.5 (1.5-5.3) |
| *≥65 years* | 27.5 (30.1) | 16.8 (9.3-33.4) | 27.3 (30.0) | 16.6 (9.1-33.2) | 28.6 (30.5) | 17.9 (10.2-34.2) | 17.1 (23.1) | 9.4 (4.5-18.8) |
| *By Charlson comorbidity index scores:* |  |  |  |  |  |  |  |  |
| *0* | 12.5 (20.7) | 5.8 (3.0-12.1) | 12.2 (20.2) | 5.7 (2.9-12.0) | 14.0 (23.1) | 6.4 (3.2-13.0) | 6.1 (12.2) | 2.9 (1.5-6.0) |
| *1* | 22.8 (28.4) | 12.8 (6.7-27.1) | 22.9 (28.5) | 12.8 (6.5-27.5) | 22.6 (28.2) | 13.0 (7.3-25.5) | 15.9 (21.8) | 8.7 (4.5-16.9) |
| *2* | 21.2 (27.3) | 11.9 (6.7-23.2) | 20.9 (27.1) | 11.7 (6.5-22.8) | 23.0 (27.9) | 13.1 (7.3-25.7) | 14.6 (20.9) | 7.7 (3.9-15.1) |
| *3* | 25.6 (29.7) | 15.0 (8.7-29.8) | 25.5 (29.9) | 14.9 (8.6-29.4) | 26.2 (28.5) | 15.5 (9.2-31.4) | 18.9 (23.1) | 11.2 (6.5-20.6) |
| *4* | 29.1 (31.5) | 17.5 (10.2-35.0) | 28.8 (31.3) | 17.1 (10.1-34.8) | 30.3 (32.4) | 18.8 (10.7-35.3) | 23.4 (26.4) | 14.2 (8.6-26.6) |
| *≥5* | 35.0 (34.5) | 23.1 (13.8-42.4) | 34.9 (34.2) | 23.0 (13.7-42.6) | 35.4 (35.8) | 23.2 (14.3-41.7) | 28.7 (29.2) | 18.4 (11.3-33.8) |

Abbreviations: Non-rCDI: non-recurrent CDI episode, rCDI: recurrent CDI episode, SD: standard deviation, IQR: interquartile range. *rCDI: Recurrent CDI was defined as having a recorded CDI episode within eight weeks from the start of the last CDI episode.

**Additional Table 4: Origin of *Clostridioides difficile* infection (CDI): Summary statistics for healthcare consumption within one year from the first infection or its proxy (controls).**

|  | **CDI**  **(**N=43,150) | | **Hospital-acquired CDI**  (N*=*39,526) | | **Community acquired CDI**  (N=3,094) | | **Control**  **(**N=355,172) | |
| --- | --- | --- | --- | --- | --- | --- | --- | --- |
|  | Mean (SD) | Median (IQR) | Mean (SD) | Median (IQR) | Mean (SD) | Median (IQR) | Mean (SD) | Median (IQR) |
| **Length of stay, in days** |  |  |  |  |  |  |  |  |
| *Total* | 27.4 (30.8) | 18.0 (7.0-37.0) | 29.5 (31.1) | 20.0 (9.0-39.0) | 2.9 (9.9) | 0.0 (0.0-0.0) | 1.6 (7.2) | 0.0 (0.0-0.0) |
| *Men* | 28.9 (31.9) | 20.0 (8.0-39.0) | 30.8 (32.2) | 21.0 (10.0-41.0) | 3.4 (11.6) | 0.0 (0.0-1.0) | 1.7 (7.6) | 0.0 (0.0-0.0) |
| *Women* | 26.1 (29.8) | 17.0 (6.0-35.0) | 28.3 (30.1) | 19.0 (8.0-37.0) | 2.6 (8.5) | 0.0 (0.0-0.0) | 1.5 (6.8) | 0.0 (0.0-0.0) |
| *≤64 years* | 24.5 (39.4) | 8.0 (2.0-31.0) | 30.2 (42.1) | 14.0 (4.0-39.0) | 1.4 (6.3) | 0.0 (0.0-0.0) | 0.3 (3.7) | 0.0 (0.0-0.0) |
| *≥65 years* | 28.4 (27.3) | 21.0 (10.0-38.0) | 29.3 (27.3) | 21.0 (11.0-39.0) | 5.7 (14.0) | 0.0 (0.0-5.0) | 2.1 (8.2) | 0.0 (0.0-0.0) |
| *By Charlson comorbidity index scores:* |  |  |  |  |  |  |  |  |
| *0* | 11.2 (18.3) | 5.0 (1.0-14.0) | 14.6 (19.8) | 8.0 (3.0-19.0) |  |  | 0.1 (1.8) | 0.0 (0.0-0.0) |
| *1* | 20.8 (25.6) | 13.0 (5.0-28.0) | 23.2 (26.1) | 15.0 (7.0-31.0) | 1.5 (4.6) | 0.0 (0.0-0.0) | 1.3 (5.8) | 0.0 (0.0-0.0) |
| *2* | 28.5 (32.0) | 19.0 (8.0-37.0) | 30.1 (32.2) | 21.0 (9.0-39.0) | 4.0 (13.1) | 0.0 (0.0-2.0) | 1.8 (7.5) | 0.0 (0.0-0.0) |
| *3* | 28.9 (30.0) | 20.0 (9.0-38.0) | 30.0 (30.1) | 21.0 (10.0-40.0) | 5.7 (15.1) | 0.0 (0.0-5.0) | 2.7 (9.0) | 0.0 (0.0-0.0) |
| *4* | 31.5 (31.3) | 23.0 (11.0-42.0) | 32.4 (31.4) | 23.0 (12.0-43.0) | 6.3 (11.2) | 0.0 (0.0-7.0) | 3.8 (11.2) | 0.0 (0.0-2.0) |
| *≥5* | 35.4 (33.8) | 26.0 (13.0-47.0) | 36.1 (33.9) | 26.0 (13.0-48.0) | 11.5 (19.0) | 2.0 (0.0-16.0) | 5.4 (13.3) | 0.0 (0.0-5.0) |
| **Number of in-hospital admissions** | |  |  |  |  |  |  |  |
| *Total* | 2.6 (1.8) | 2.0 (1.5-3) | 2.7 (1.8) | 2.3 (1.5-3.3) | 1.4 (0.9) | 1.0 (1.0-1.5) | 1.3 (0.7) | 1.0 (1.0-1.5) |
| *Men* | 2.7 (1.8) | 2.3 (1.5-3.3) | 2.8 (1.8) | 2.3 (1.7-3.4) | 1.5 (1.0) | 1.0 (1.0-1.5) | 1.4 (0.7) | 1.0 (1.0-1.5) |
| *Women* | 2.5 (1.7) | 2.0 (1.3-3.0) | 2.6 (1.7) | 2.0 (1.5-3) | 1.4 (0.8) | 1.0 (1.0-1.5) | 1.4 (0.6) | 1.0 (1.0-1.4) |
| *≤64 years* | 2.6 (2.5) | 2.0 (1.0-3.1) | 3.0 (2.6) | 2.0 (1.2-3.7) | 1.3 (0.8) | 1.0 (1.0-1.0) | 1.1 (0.5) | 1.0 (1.0-1.0) |
| *≥65 years* | 2.6 (1.5) | 2.2 (1.6-3.0) | 2.6 (1.5) | 2.3 (1.7-3.1) | 1.7 (1.0) | 1.3 (1.0-2.0) | 1.4 (0.7) | 1.0 (1.0-1.7) |
| *By Charlson comorbidity index scores:* |  |  |  |  |  |  |  |  |
| *0* | 1.5 (0.9) | 2.0 (1.0-2.0) | 1.6 (1.0) | 1.0 (1.0-2.0) | 1.1 (0.4) | 1.0 (1.0-1.0) | 1.0 (0.2) | 1.0 (1.0-1.0) |
| *1* | 2.0 (1.2) | 1.7 (1.0-2.5) | 2.1 (1.2) | 2.0 (1.2-2.5) | 1.3 (0.7) | 1.0 (1.0-1.4) | 1.3 (0.6) | 1.0 (1.0-1.5) |
| *2* | 2.9 (2.3) | 2.3 (1.5-3.5) | 3.0 (2.4) | 2.3 (1.6-3.5) | 1.7 (1.1) | 1.2 (1.0-2.0) | 1.5 (0.8) | 1.0 (1.0-1.7) |
| *3* | 2.7 (1.8) | 2.2 (1.6-3.2) | 2.7 (1.8) | 2.3 (1.7-3.3) | 1.9 (1.1) | 1.5 (1.0-2.3) | 1.6 (0.8) | 1.3 (1.0-2.0) |
| *4* | 2.8 (1.7) | 2.3 (1.8-3.3) | 2.8 (1.7) | 2.4 (1.8-3.3) | 1.9 (1.2) | 1.5 (1.1-2.3) | 1.8 (0.9) | 2.0 (1.5-2.0) |
| *≥5* | 3.1 (1.6) | 2.7 (2.0-3.7) | 3.1 (1.6) | 2.8 (2.0-3.7) | 2.4 (1.2) | 2.1 (1.5-2.8) | 1.9 (0.9) | 1.8 (1.3-2.3) |
| **Number of outpatient care visits** | |  |  |  |  |  |  |  |
| *Total)* | 4.0 (10.2) | 2.0 (1.0-3.4) | 4.1 (10.5) | 2.0 (1.0-3.5) | 2.7 (5.2) | 2.0 (1.0-2.8) | 1.9 (2.6) | 1.3 (1.0-2.0) |
| *Men* | 4.7 (12.3) | 2.0 (1.0-3.8) | 4.8 (12.6) | 2.0 (1.0-3.8) | 3.0 (6.4) | 2.0 (1.0-3.0) | 2.0 (3.1) | 1.5 (1.0-2.0) |
| *Women* | 3.4 (7.9) | 2.0 (1.0-3.1) | 3.5 (8.2) | 2.0 (1.0-3.2) | 2.6 (4.2) | 1.9 (1.2-2.7) | 1.8 (2.2) | 1.3 (1.0-2.0) |
| *≤64 years* | 5.2 (11.2) | 2.3 (1.3-2.0) | 5.8 (12.2) | 2.5 (1.3-5.5) | 2.7 (5.1) | 2.0 (1.0-2.8) | 1.9 (2.4) | 1.1 (1.0-2.0) |
| *≥65 years* | 3.6 (9.8) | 2.0 (1.0-3.0) | 3.6 (10.0) | 2.0 (1.0-3.0) | 2.8 (5.3) | 2.0 (1.3-2.9) | 1.9 (2.7) | 1.3 (1.0-2.0) |
| *By Charlson comorbidity index scores:* |  |  |  |  |  |  |  |  |
| *0* | 1.8 (1.8) | 1.0 (1.0-2.0) | 1.7 (1.9) | 1.5 (1-2.3) | 2.0 (1.5) | 1.5 (1.0-2.3) | 1.3 (0.7) | 1.0 (1.0-1.4) |
| *1* | 1.8 (1.5) | 1.3 (1.0-2.0) | 1.7 (1.4) | 1.0 (1.0-2.0) | 2.2 (1.6) | 1.8 (1.3-2.5) | 1.4 (0.8) | 1.0 (1.0-1.5) |
| *2* | 4.1 (7.0) | 2.0 (1.0-4.5) | 4.2 (6.9) | 2.0 (1-4.5) | 3.7 (7.8) | 2.3 (1.5-3.7) | 2.3 (2.7) | 1.5 (1.0-2.3) |
| *3* | 3.6 (7.8) | 2.0 (1.3-3.5) | 3.6 (8.0) | 2.0 (1.2-3.5) | 3.3 (3.8) | 2.3 (1.6-3.5) | 2.2 (2.5) | 1.7 (1.0-2.3) |
| *4* | 4.0 (9.8) | 2.0 (1.3-3.5) | 4.0 (9.8) | 2.0 (1.3-3.5) | 3.9 (9.9) | 2.5 (1.8-3.5) | 2.2 (3.5) | 1.7 (1.2-2.8) |
| *≥5* | 6.3 (16.1) | 2.6 (1.7-4.4) | 6.3 (16.2) | 2.6 (1.7-4.3) | 5.4 (11.6) | 2.8 (2.0-4.9) | 2.6 (5.1) | 2.0 (1.4-2.7) |
| **Number of dispensed prescriptions** | |  |  |  |  |  |  |  |
| *Total* | 25.5 (30.5) | 14.8 (7.7-30.0) | 26.6 (31.0) | 15.7 (8.3-31.5) | 13.0 (19.8) | 6.8 (3.0-14.4) | 13.7 (21.1) | 6.7 (2.8-14.7) |
| *Men* | 22.6 (27.3) | 13.6 (7.3-25.9) | 23.4 (27.7) | 14 (7.8-26.7) | 11.7 (17.8) | 6.2 (2.7-13.9) | 11.8 (18.2) | 6.3 (2.6-12.9) |
| *Women* | 27.9 (32.8) | 16.3 (8.1-34.0) | 29.3 (33.4) | 17.4 (8.9-36.2) | 13.8 (20.9) | 7.0 (3.3-15.0) | 15.3 (23.1) | 7.1 (2.9-16.6) |
| *≤64 years* | 19.5 (30.9) | 9.1 (4.2-20.3) | 21.7 (32.9) | 10.6 (5-32.1) | 10.1 (17.5) | 4.6 (2.5-10.5) | 5.5 (11.5) | 2.5 (1.5-5.3) |
| *≥65 years* | 27.5 (30.1) | 16.8 (9.3-33.4) | 27.9 (30.4) | 17.1 (9.4-33.9) | 18.7 (22.5) | 11.7 (6.4-21.3) | 17.1 (23.1) | 9.4 (4.5-18.8) |
| *By Charlson comorbidity index scores:* |  |  |  |  |  |  |  |  |
| *0* | 12.5 (20.7) | 5.8 (3.0-12.1) | 14.3 (22.5) | 6.8 (3.4-14.5) | 6.9 (12.5) | 3.6 (2.3-7.1) | 6.1 (12.2) | 2.9 (1.5-6.0) |
| *1* | 22.8 (28.4) | 12.8 (6.7-27.1) | 24.0 (29.0) | 13.6 (7.2-28.9) | 13.9 (21.8) | 7.8 (3.7-14.9) | 15.9 (21.8) | 8.7 (4.5-16.9) |
| *2* | 21.2 (27.3) | 11.9 (6.7-23.2) | 21.5 (27.5) | 12 (6.8-23.7) | 16.3 (21.5) | 9.4 (4.9-17.4) | 14.6 (20.9) | 7.7 (3.9-15.1) |
| *3* | 25.6 (29.7) | 15.0 (8.7-29.8) | 25.9 (30.0) | 15.2 (8.7-30.2) | 20.7 (24.7) | 13.3 (8.8-22.2) | 18.9 (23.1) | 11.2 (6.5-20.6) |
| *4* | 29.1 (31.5) | 17.5 (10.2-35.0) | 29.3 (31.8) | 17.6 (10.2-35.5) | 21.2 (20.2) | 15.8 (9.9-25.4) | 23.4 (26.4) | 14.2 (8.6-26.6) |
| *≥5* | 35.0 (34.5) | 23.1 (13.8-42.4) | 35.1 (34.6) | 23.1 (13.8-42.7) | 29.3 (25.9) | 21.4 (13.5-34.4) | 28.7 (29.2) | 18.4 (11.3-33.8) |

Abbreviations: Non-rCDI: non-recurrent CDI episode, rCDI: recurrent CDI episode, SD: standard deviation, IQR: interquartile range. *rCDI: Recurrent CDI was defined as having a recorded CDI episode within eight weeks from the start of the last CDI episode.

**Additional Table 5:** ***Clostridioides difficile* infection (CDI) and recurrence: descriptive statistics for total length of in-hospital stay (LOS) and total number of hospital admissions, stratified by sex, age, Charlson comorbidity index score, and likely origin (Total study period 2006-2019).**

|  | **CDI (N=43,150)** | | **Non-recurrent CDI (N=35,899)** | | **Recurrent CDI (N=7,251)** | | **Control (N=355,172)** | |
| --- | --- | --- | --- | --- | --- | --- | --- | --- |
|  | Mean (SD) | Median (IQR) | Mean (SD) | Median (IQR) | Mean (SD) | Median (IQR) | Mean (SD) | Median (IQR) |
| **Total Length of stay, in days** |  |  |  |  |  |  |  |  |
| *Total* | 73.6 (75.3) | 54 (24-99) | 71.3 (73.5) | 52 (23-96) | 84.5 (82.7) | 63 (29-114) | 17.5 (35.0) | 3 (0-22) |
| *Men* | 77.8 (75.8) | 58 (28-105) | 75.3 (73.7) | 56 (27-102) | 90.8 (85.2) | 69 (35-120) | 18.5 (38.2) | 4 (0-24) |
| *Women* | 70.0 (74.7) | 50 (20-94) | 67.9 (73.2) | 49 (20-92) | 79.6 (80.5) | 59 (24-107) | 16.6 (32.2) | 3 (0-20) |
| *≤64 years* | 68.8 (102.3) | 27 (5-95) | 67.3 (100.2) | 27 (4-94) | 76.6 (112.9) | 28 (6-98) | 4.2 (23.8) | 0 (0-1) |
| *≥65 years* | 75.2 (63.6) | 59 (32-100) | 72.7 (61.7) | 57 (31-97) | 86.9 (70.8) | 69 (39-116) | 23.2 (37.5) | 10 (0-32) |
| *Hospital acquired* | 78.2 (75.7) | 58 (29-104) | 75.8 (73.9) | 57 (28-101) | 90.1 (83.0) | 69 (35-119) | - | - |
| *Community acquired* | 17.6 (40.0) | 3 (0-17) | 16.3 (38.2) | 1 (0-15) | 23.5 (46.8) | 5 (0-28) | - | - |
| *By Charlson comorbidity index scores:* |  |  |  |  |  |  |  |  |
| *0* | 20.1 (38.3) | 8 (2-25) | 19.8 (38.9) | 7 (2-25) | 22.2 (35.1) | 9 (3-28) | 1.8 (7.5) | 0 (0-0) |
| *1* | 44.7 (52.0) | 31 (13-59) | 43.4 (48.5) | 31 (12-57) | 51.7 (67.3) | 36.5 (14-66) | 14.4 (29.9) | 6 (1-18) |
| *2* | 65.0 (61.0) | 49 (25-86) | 63.2 (59.6) | 48 (25-84) | 72.2 (67.3) | 54 (27-94) | 19.0 (29.0) | 9 (1-26) |
| *3* | 73.3 (63.9) | 56 (31-95) | 71.4 (61.7) | 55 (30-94) | 83.1 (73.4) | 65 (37-104) | 29.8 (42.5) | 19 (7-40) |
| *4* | 86.7 (71.8) | 69 (42-110) | 84.3 (70.1) | 68 (41-107) | 98.0 (78.2) | 78 (48-128) | 41.0 (47.4) | 30 (14-55) |
| *≥5* | 115.5 (89.7) | 93 (58-147) | 112.7 (88.7) | 91 (56-144) | 127.9 (92.9) | 105 (66-165) | 61.3 (55.0) | 47 (25-82) |
| **Total number of in-hospital admissions** |  |  |  |  |  |  |  |  |
| *Total* | 9.1 (9.4) | 7 (3-12) | 2.9 (4.8) | 1 (0-4) | 11.3 (11.1) | 8 (5-15) | 8.7 (8.9) | 6 (3-11) |
| *Men* | 9.7 (9.4) | 7 (4-13) | 9.2 (9.0) | 7 (3-12) | 12.2 (11.1) | 10 (5-16) | 3.2 (5.0) | 1 (0-4) |
| *Women* | 8.7 (9.3) | 6 (3-11) | 8.2 (8.8) | 6 (3-11) | 10.6 (11.1) | 8 (4-14) | 2.7 (4.6) | 1 (0-4) |
| *≤64 years* | 9.0 (12.5) | 4 (1-12) | 8.7 (11.8) | 4 (1-12) | 10.9 (15.9) | 5 (2-14) | 0.8 (2.7) | 0 (0-1) |
| *≥65 years* | 9.1 (8.0) | 7 (4-12) | 8.7 (7.7) | 7 (4-11) | 11.5 (9.2) | 9 (5-15) | 3.8 (5.2) | 2 (0-5) |
| *Hospital acquired* | 9.6 (9.4) | 7 (4-12) | 9.1 (9.0) | 7 (3-12) | 12.0 (11.2) | 9 (5-15) | - | - |
| *Community acquired* | 3.0 (5.8) | 1 (0-4) | 2.6 (5.5) | 1 (0-3) | 3.9 (6.8) | 2 (0-5) | - | - |
| *By Charlson comorbidity index scores:* | |  |  |  |  |  |  |  |
| *0* | 2.6 (3.9) | 1 (1-3) | 2.5 (3.7) | 1 (1-3) | 3.5 (4.8) | 2 (1-4) | 0.4 (1.2) | 0 (0-0) |
| *1* | 5.3 (6.7) | 4 (2-7) | 5.0 (5.5) | 3 (2-6) | 7.1 (11.1) | 5 (3-8) | 2.4 (3.0) | 2 (1-3) |
| *2* | 8.0 (7.6) | 6 (3-10) | 7.6 (7.4) | 6 (3-10) | 9.7 (8.4) | 7 (4-12) | 3.2 (4.0) | 2 (1-4) |
| *3* | 8.8 (7.3) | 7 (4-11) | 8.4 (7.1) | 7 (4-11) | 10.5 (7.9) | 8 (5-13) | 4.8 (4.6) | 4 (2-6) |
| *4* | 10.6 (8.8) | 8 (5-13) | 10.1 (8.4) | 8 (5-12) | 12.9 (10.2) | 10 (7-16) | 6.7 (5.6) | 5 (3-9) |
| *≥5* | 14.7 (11.4) | 12 (7-18) | 14.1 (10.9) | 11 (7-18) | 17.2 (13.1) | 14 (9-21) | 9.9 (8.1) | 8 (5-13) |
| **Total number of outpatient visits** |  |  |  |  |  |  |  |  |
| *Total* | 23.2 (83.1) | 7 (2-19) | 22.3 (80.8) | 7 (2-18) | 27.4 (93.7) | 9 (3-22) | 8.7 (25.6) | 4 (1-9) |
| *Men* | 28.3 (102.1) | 8 (3-21) | 26.7 (97.0) | 8 (2-20) | 36.7 (125.7) | 10 (4-24) | 9.8 (31.3) | 4 (1-11) |
| *Women* | 18.8 (62.0) | 6 (2-17) | 18.5 (63.0) | 6 (2-17) | 20.3 (57.1) | 8 (3-28) | 7.8 (20.0) | 3 (1-8) |
| *≤64 years* | 32.5 (99.2) | 10 (3-30) | 32.4 (101.3) | 9 (3-30) | 32.7 (87.1) | 11 (44-31) | 7.5 (18.9) | 3 (1-7) |
| *≥65 years* | 19.9 (76.4) | 6 (2-16) | 18.7 (71.7) | 6 (2-16) | 25.8 (95.7) | 8 (3-20) | 9.0 (27.1) | 4 (1-10) |
| *Hospital acquired* | 23.7 (85.5) | 7 (2-19) | 22.9 (83.3) | 7 (2-19) | 28.0 (95.9) | 9 (3-22) |  |  |
| *Community acquired* | 15.6 (50.7) | 6 (2-15) | 14.7 (44.4) | 5 (2-144) | 19.5 (72.4) | 8 (4-18) |  |  |
| *By Charlson comorbidity index scores:* |  |  |  |  |  |  |  |  |
| *0* | 6.2 (11.8) | 2 (1-6) | 5.9 (11.7) | 2 (1-5) | 7.9 (12.7) | 4 (1-8) | 3.0 (4.6) | 2 (1-3) |
| *1* | 7.4 (14.4) | 3 (1-8) | 7.0 (13.6) | 3 (1-7) | 9..4 (18.0) | 4 (1-9) | 4.3 (7.9) | 2 (1-5) |
| *2* | 17.7 (45.3) | 7 (2-19) | 17.2 (44.9) | 7 (2-19) | 20.5 (47.5) | 9 (3-21) | 9.0 (16.7) | 5 (2-11) |
| *3* | 18.1 (57.8) | 7 (3-18) | 17.9 (59.4) | 7 (3-18) | 19.2 (49.3) | 9 (4-20) | 11.2 (23.2) | 6 (3-14) |
| *4* | 23.8 (83.1) | 9 (3-21) | 22.9 (80.7) | 8 (3-20) | 27.9 (93.5) | 10 (4-25) | 13.2 (32.6) | 8 (3-15) |
| *≥5* | 45.1 (133.7) | 14 (6-32) | 43.8 (130.2) | 14 (6-31) | 50.7 (148.3) | 15 (7-35) | 20.8 (58.4) | 11 (5-22) |
| **Total number of prescriptions** |  |  |  |  |  |  |  |  |
| *Total* | 262.7 (355.1) | 146 (61-316) | 257.2 (350.1) | 141 (58-309 | 290.1 (377.3) | 169.5 (75-348) | 15.66 (50.7) | 6 (2-15) |
| *Men* | 226.3 (315.9) | 126 (54-126) | 221.6 (311.6) | 122 (52-260) | 250.8 (336.8) | 148 (69-297) | 127.1 (207.7) | 64 (14-155) |
| *Women* | 293.5 (382.3) | 165 (69-363) | 287.8 (377.6) | 160 (66-354) | 320.2 (403.0) | 187 (82-399) | 169.2 (262.4) | 82 (21-201) |
| *≤64 years* | 223.3 (405.5) | 89 (30-230) | 219.6 (398.6) | 87 (30-227) | 243.1 (440.6) | 100 (33-249.5) | 61.4 (162.6) | 15 (5-58) |
| *≥65 years* | 276.0 (335.3) | 165 (76-341) | 270.1 (330.9) | 160 (74-334) | 304.5 (354.4) | 189 (93-375) | 187.2 (257.1) | 108 (43-223) |
| *Hospital acquired* | 271.4 (360.4) | 152 (65-328) | 265.6 (355.8) | 147 (63-320) | 300.6 (381.2) | 176 (81-365) |  |  |
| *Community acquired* | 157.4 (257.6) | 75 (22-191) | 152.2 (251.0) | 73 (21-184) | 180.7 (284.2) | 85 (27-211) |  |  |
| *By Charlson comorbidity index scores:* |  |  |  |  |  |  |  |  |
| *0* | 134.0 (254.3) | 55 (18-136) | 129.2 (246.4) | 54 (18-132) | 160.1 (292.8) | 65 (23-158) | 64.1 (142.0) | 19 (5-70) |
| *1* | 229.8 (323.4) | 125 (52-276) | 226.1 (317.1) | 123 (52-272) | 249.7 (354.8) | 141.5 (60-292) | 170.5 (238.1) | 101 (41-200) |
| *2* | 207.9 (317.5) | 102 (44-234) | 201.4 (311.9) | 99 (42-224) | 241.2 (343.3) | 126 (59-284) | 157.3 (233.5) | 85 (32-183) |
| *3* | 259.1 (346.1) | 146 (70-304) | 255.1 (347.6) | 142 (67-297) | 279.1 (338.1) | 167 (82-335) | 210.5 (262.5) | 132 (65-248) |
| *4* | 303.3 (371.2) | 177 (87-366) | 298.2 (367.7) | 174 (86-359) | 327.4 (386.8) | 196 (98-391) | 262.5 (302.0) | 169 (91-313) |
| *≥5* | 368.2 (401.7) | 241 (128-453) | 364.5 (395.7) | 239 (124-449) | 384.7 (427.0) | 250 (138-468) | 329.3 (343.7) | 223 (125-399) |

Abbreviations: Non-rCDI: non-recurrent CDI episode, rCDI: recurrent CDI episode, SD: standard deviation, IQR: interquartile range.

rCDI: Recurrent CDI was defined as having a recorded CDI episode within eight weeks from the start of the last CDI episode.

Age for controls corresponds to the age of their matched case at the time of their first recorded CDI episode.

**Additional Table 6: Origin of the *Clostridioides difficile* infection (CDI): Descriptive statistics for total length of in-hospital stay (LOS) and total number of hospital admissions, stratified by sex, age, Charlson comorbidity index score (Total study period: 2006-2019)**

|  | **CDI (N=43,150)** | | **Hospital-acquired CDI**  (N*=*39,526) | | **Community acquired CDI**  (N=3,094) | | **Control (N=355,172)** | |
| --- | --- | --- | --- | --- | --- | --- | --- | --- |
|  | Mean (SD) | Median (IQR) | Mean (SD) | Median (IQR) | Mean (SD) | Median (IQR) | Mean (SD) | Median (IQR) |
| **Total length of stay, in days** |  |  |  |  |  |  |  |  |
| *Total* | 73.6 (75.3) | 54 (24-99) | 78.2 (75.7) | 58 (29-104) | 17.9 (40.3) | 2 (0-17) | 17.5 (35) | 3 (0-22) |
| *Men* | 77.8 (75.8) | 58 (28-105) | 81.8 (76.0) | 62 (32-108) | 19.0 (40.3) | 2 (0-19) | 18.5 (38.2) | 4 (0-24) |
| *Women* | 70.0 (74.7) | 50 (20-94) | 75.0 (75.3) | 55 (26-100) | 16.7 (39.7) | 2 (0-15) | 16.6 (32.2) | 3 (0-20) |
| *≤64 years* | 68.8 (102.3) | 27 (5-95) | 83.1 (108.4) | 45 (11-116) | 10.7 (34.9) | 0 (0-5) | 4.2 (23.8) | 0 (0-1) |
| *≥65 years* | 75.2 (63.6) | 59 (32-100) | 76.8 (63.6) | 61 (33-102) | 30.9 (45.4) | 16 (2-42) | 23.2 (37.5) | 10 (0-32) |
| *By Charlson comorbidity index scores:* |  |  |  |  |  |  |  |  |
| *0* | 20.1 (38.3) | 8 (2-25) | 25.7 (42.3) | 13 (5-32) | 3.1 (10.4) | 0 (0-1) | 1.8 (7.5) | 0 (0-0) |
| *1* | 44.7 (52.0) | 31 (13-59) | 48.9 (52.7) | 36 (17-63) | 10.6 (27.1) | 3 (0-11) | 14.4 (29.9) | 6 (1-18) |
| *2* | 65.0 (61.0) | 49 (25-86) | 67.6 (61.0) | 51 (28-89) | 21.3 (34.4) | 8 (1-24.5) | 19.0 (29.0) | 9 (1-26) |
| *3* | 73.3 (63.9) | 56 (31-95) | 75.0 (64.2) | 58 (33-97) | 36.3 (47.0) | 23 (8-50) | 29.8 (42.5) | 19 (7-40) |
| *4* | 86.7 (71.8) | 69 (42-110) | 87.9 (71.7) | 70 (43-111) | 53.2 (72.2) | 32 (11-62) | 41.0 (47.4) | 30 (14-55) |
| *≥5* | 115.5 (89.7) | 93 (58-147) | 116.4 (89.8) | 94 (59-148) | 75.2 (69.0) | 56 (29-100) | 61.3 (55.0) | 47 (25-82) |
| **Total number of in-hospital admissions** |  |  |  |  |  |  |  |  |
| *Total* | 9.1 (9.4) | 7 (3-12) | 9.6 (9.4) | 7 (4-12) | 3.0 (5.8) | 1 (0-4) | 8.7 (8.9) | 6 (3-11) |
| *Men* | 9.7 (9.4) | 7 (4-13) | 10.1 (9.4) | 8 (4-13) | 3.4 (6.4) | 1 (0-4) | 3.2 (5.0) | 1 (0-4) |
| *Women* | 8.7 (9.3) | 6 (3-11) | 9.2 (9.4) | 7 (3-12) | 2.8 (5.3) | 1 (0-3) | 2.7 (4.6) | 1 (0-4) |
| *≤64 years* | 9.0 (12.5) | 4 (1-12) | 10.7 (13.2) | 6 (2-14) | 2.1 (5.0) | 0 (0-2) | 0.8 (2.7) | 0 (0-1) |
| *≥65 years* | 9.1 (8.0) | 7 (4-12) | 9.3 (8.0) | 7 (4-12) | 5.0 (6.6) | 3 (1-7) | 3.8 (5.2) | 2 (0-5) |
| *By Charlson comorbidity index scores:* | |  |  |  |  |  |  |  |
| *0* | 2.6 (3.9) | 1 (1-3) | 3.2 (4.2) | 2 (1-4) | 0.7 (2.0) | 0 (0-1) | 0.4 (1.2) | 0 (0-0) |
| *1* | 5.3 (6.7) | 4 (2-7) | 5.7 (6.9) | 4 (2-7) | 2.2 (4.2) | 1 (0-3) | 2.4 (3.0) | 2 (1-3) |
| *2* | 8.0 (7.6) | 6 (3-10) | 8.2 (7.6) | 6 (4-10) | 3.8 (4.8) | 2 (1-5) | 3.2 (4.0) | 2 (1-4) |
| *3* | 8.8 (7.3) | 7 (4-11) | 8.9 (7.3) | 7 (4-11) | 5.6 (5.2) | 4 (2-8) | 4.8 (4.6) | 4 (2-6) |
| *4* | 10.6 (8.8) | 8 (5-13) | 10.6 (8.8) | 8 (5-13) | 8.0 (7.9) | 5 (3-11) | 6.7 (5.6) | 5 (3-9) |
| *≥5* | 14.7 (11.4) | 12 (7-18) | 14.7 (11.4) | 12 (7-19) | 12.4 (10.7) | 9 (6-16) | 9.9 (8.1) | 8 (5-13) |
| **Total number of outpatient visits** |  |  |  |  |  |  |  |  |
| *Total* | 23.2 (83.1) | 7 (2-19) | 23.7 (85.5) | 7 (2-19) | 15.6 (50.7) | 6 (2-15) | 8.7 (25.6) | 4 (1-9) |
| *Men* | 28.3 (102.1) | 8 (3-21) | 28.9 (104.5) | 8 (3-21) | 17.7 (67.4) | 5 (2-15) | 9.8 (31.3) | 4 (1-11) |
| *Women* | 18.8 (62.0) | 6 (2-17) | 19.2 (64.2) | 6 (2-17) | 14.2 (36.0) | 6 (3-15) | 7.8 (20.0) | 3 (1-8) |
| *≤64 years* | 32.5 (99.2) | 10 (3-30) | 36.9 (108.8) | 12 (3-35) | 14.1 (43.5) | 5 (2-13) | 7.5 (18.9) | 3 (1-7) |
| *≥65 years* | 19.9 (76.4) | 6 (2-16) | 19.9 (77.1) | 66 (2-16) | 18.3 (62.2) | 8 (3-19) | 9.0 (27.1) | 4 (1-10) |
| *By Charlson comorbidity index scores:* |  |  |  |  |  |  |  |  |
| *0* | 6.2 (11.8) | 2 (1-6) | 6.0 (11.9) | 2 (1-5) | 6.5 (10.9) | 3 (1-6) | 3.0 (4.6) | 2 (1-3) |
| *1* | 7.4 (14.4) | 3 (1-8) | 6.7 (13.7) | 2 (1-7) | 11.8 (17.8) | 7 (3-13) | 4.3 (7.9) | 2 (1-5) |
| *2* | 17.7 (45.3) | 7 (2-19) | 17.4 (44.5) | 7 (2-19) | 20.9 (59.1) | 10.5 (5-22) | 9.0 (16.7) | 5 (2-11) |
| *3* | 18.1 (57.8) | 7 (3-18) | 17.8 (58.6) | 7 (3-18) | 22.3 (30.8) | 13 (6-27) | 11.2 (23.2) | 6 (3-14) |
| *4* | 23.8 (83.1) | 9 (3-21) | 23.4 (81.5) | 9 (3-20) | 35.0 (130.9) | 16 (8-32) | 13.2 (32.6) | 8 (3-15) |
| *≥5* | 45.1 (133.7) | 14 (6-32) | 44.8 (134.6) | 13 (6-31) | 51.7 (108.1) | 27 (12-56) | 20.8 (58.4) | 11 (5-22) |
| ***Total number of prescriptions*** |  |  |  |  |  |  |  |  |
| *Total* | 262.7 (355.1) | 146 (61-316) | 157.4 (257.6) | 75 (22-191) | 271.4 (360..4) | 152 (65-328) | 15.66 (50.7) | 6 (2-15) |
| *Men* | 226.3 (315.9) | 126 (54-126) | 232.5 (321.2) | 130 (57-273) | 131.2 (203.7) | 62 (17-172) | 127.1 (207.7) | 64 (14-155) |
| *Women* | 293.5 (382.3) | 165 (69-363) | 305.0 (388.0) | 174 (74-377) | 174.1 (205.6) | 82 (26-200) | 169.2 (262.4) | 82 (21-201) |
| *≤64 years* | 223.3 (405.5) | 89 (30-230) | 246.8 (429.3) | 101 (36-259) | 124.1 (250.2) | 45 (15-135) | 61.4 (162.6) | 15 (5-58) |
| *≥65 years* | 276.0 (335.3) | 165 (76-341) | 278.3 (338.3) | 166 (77-344) | 221 (259.6) | 142 (69-269) | 187.2 (257.1) | 108 (43-223) |
| *By Charlson comorbidity index scores:* |  |  |  |  |  |  |  |  |
| *0* | 134.0 (254.3) | 55 (18-136) | 153.0 (276.2) | 66 (22-157) | 75.9 (156.6) | 30 (12-80) | 64.1 (142.0) | 19 (5-70) |
| *1* | 229.8 (323.4) | 125 (52-276) | 237.3 (328.7) | 129 (55-286) | 173.1 (279.9) | 98 (29.5-202) | 170.5 (238.1) | 101 (41-200) |
| *2* | 207.9 (317.5) | 102 (44-234) | 208.1 (319.1) | 101 (44-234) | 205.6 (285.9) | 119 (48-232) | 157.3 (233.5) | 85 (32-183) |
| *3* | 259.1 (346.1) | 146 (70-304) | 259.9 (348.0) | 145 (68-305) | 262.0 (348.7) | 164.0 (99.0-306.0) | 210.5 (262.5) | 132 (65-248) |
| *4* | 303.3 (371.2) | 177 (87-366) | 304.5 (374.6) | 176 (87-368) | 265.4 (261.4) | 205.0 (114.0-329.0) | 262.5 (302.0) | 169 (91-313) |
| *≥5* | 368.2 (401.7) | 241 (128-453) | 368.4 (401.5) | 240 (127-454) | 358.1 (326.7) | 269.0 (175.0-429.0) | 329.3 (343.7) | 223 (125-399) |

**Additional Table 7: Total burden of *Clostridioides difficile* infection (CDI) in terms of number of patients, length of stay and number of hospitalisations during the first year and during the entire study period**

|  |  | **Within first year after first CDI episode** | | **Total study period** | |
| --- | --- | --- | --- | --- | --- |
|  |  | ***Length of stay*** | ***Hospitalisations*** | ***Length of stay*** | ***Hospitalisations*** |
|  | ***N (%)*** | ***Sum (%)*** | ***Sum (%)*** | ***Sum (%)*** | ***Sum (%)*** |
| *Total CDI group* | 43,150 (100.0) | 1,177,761 (100.0) | 111,494 (100.0) | 3,173,513 (100.0) | 393,290 (100.0) |
| *<65 years* | 10,884 (25.2) | 264,515 (22.5) | 28,425 (25.5) | 748,525 (23.6) | 98,256 (25.0) |
| *>= 65 years* | 32,266 (74.8) | 913,246 (77.5) | 83,069 (74.5) | 2,424,988 (76.4) | 295,034 (75.0) |
| *Hospital-acquired CDI* | 39,526 (91.6) | 1,164,323 (98.9) | 106,126 (95.2) | 3,089,647 (97.4) | 379,303 (96.4) |
| *Community-acquired CDI* | 3,094 (7.2) | 8,582 (0.7) | 4,175 (3.7) | 54,462 (1.7) | 9,432 (2.4) |
| *Non-recurrent CDI* | 35,899 (83.2) | 946,782 (80.4) | 89,610 (80.4) | 2,561,116 (80.7) | 311,202 (79.1) |
| *Recurrent CDI* | 7,251 (16.8) | 230,980 (19.6) | 21,884 (19.6) | 612,397 (19.3) | 82,088 (20.9) |
| *Charlson comorbidity score* |  |  |  |  |  |
| *0* | 6,126 (14.2) | 67,784 (5.8) | 9,136 (8.2) | 123,345 (3.9) | 16,016 (4.1) |
| *1* | 5,321 (12.3) | 109,961 (9.3) | 10,535 (9.4) | 237,729 (7.5) | 28,370 (7.2) |
| *2* | 8,090 (18.8) | 229,779 (19.5) | 23,506 (21.1) | 525,991 (16.6) | 64,370 (16.4) |
| *3* | 6,846 (15.9) | 197,518 (16.8) | 18,413 (16.5) | 501,849 (15.8) | 59,893 (15.2) |
| *4* | 5,261 (12.2) | 165,455 (14.0) | 14,596 (13.1) | 456,003 (14.4) | 55,509 (14.1) |
| *>=5* | 11,506 (26.7) | 407,265 (34.6) | 35,309 (31.7) | 1,328,596 (41.9) | 169,132 (43.0) |
